# m^1^A inhibition fuels oncolytic virus-elicited antitumor immunity via downregulating MYC/PD-L1 signaling

**DOI:** 10.1038/s41368-024-00304-0

**Published:** 2024-05-10

**Authors:** Shujin Li, Tian Feng, Yuantong Liu, Qichao Yang, An Song, Shuo Wang, Jun Xie, Junjie Zhang, Bifeng Yuan, Zhijun Sun

**Affiliations:** 1 State Key Laboratory of Oral & Maxillofacial Reconstruction and Regeneration, Key Laboratory of Oral Biomedicine Ministry of Education, Hubei Key Laboratory of Stomatology, School & Hospital of Stomatology, Frontier Science Center for Immunology and Metabolism, Taikang Center for Life and Medical Sciences, Wuhan University, Wuhan, China; 2 School of Public Health, Wuhan University, Wuhan, China; 3 State Key Laboratory of Virology, Medical Research Institute, Wuhan University, Wuhan, China; 4 Department of Oral Maxillofacial-Head Neck Oncology, School and Hospital of Stomatology, Wuhan University, Wuhan, China

**Keywords:** Oral cancer, Cancer microenvironment, Cancer therapy

## Abstract

*N*^1^-methyladenosine (m^1^A) RNA methylation is critical for regulating mRNA translation; however, its role in the development, progression, and immunotherapy response of head and neck squamous cell carcinoma (HNSCC) remains largely unknown. Using *Tgfbr1* and *Pten* conditional knockout (2cKO) mice, we found the neoplastic transformation of oral mucosa was accompanied by increased m^1^A modification levels. Analysis of m^1^A-associated genes identified TRMT61A as a key m^1^A writer linked to cancer progression and poor prognosis. Mechanistically, TRMT61A-mediated tRNA-m^1^A modification promotes MYC protein synthesis, upregulating programmed death-ligand 1 (PD-L1) expression. Moreover, m^1^A modification levels were also elevated in tumors treated with oncolytic herpes simplex virus (oHSV), contributing to reactive PD-L1 upregulation. Therapeutic m^1^A inhibition sustained oHSV-induced antitumor immunity and reduced tumor growth, representing a promising strategy to alleviate resistance. These findings indicate that m^1^A inhibition can prevent immune escape after oHSV therapy by reducing PD-L1 expression, providing a mutually reinforcing combination immunotherapy approach.

## Introduction

Head and neck cancer accounts for roughly 4.5% of the newly diagnosed cancer cases and about 45 000 deaths worldwide in 2020.^1^ The majority of cancers arising in the head and neck originate from the mucosal epithelium lining the oral cavity, pharynx, and larynx, collectively termed head and neck squamous cell carcinoma (HNSCC).^2^ HNSCC accounts for the majority of primary head and neck cancer,^2^ with a 5-year survival rate of less than 70%.^3^ This condition underscores the urgent need to elucidate the key pathways promoting HNSCC progression and to develop therapies for HNSCC patients.

A defining characteristic of cancer cells is their increased proliferative capacity, necessitating elevated protein production.^4^ RNA modifications play a critical role in post-transcriptional regulation of gene expression. One evolutionarily conserved epitranscriptomic mark is *N*^1^-methyladenosine (m^1^A), catalyzed by the tRNA methyltransferase complex comprising the RNA-binding component TRMT6 and the catalytic component TRMT61A.^5^ The TRMT6/61A complex is located in the cytosol and seems to recognize targets in a structure-dependent manner, methylating both cyto-tRNA m^1^A58 (T-loop) and mRNAs with a T-loop-like structure.^6^ Studies report that TRMT6/TRMT61A-mediated m^1^A58 modification of tRNA enhances translation initiation and elongation.^7^ Maintaining RNA structure/folding is considered the most important m^1^A function, especially in tRNAs that regulate protein biosynthesis.^6^ To date, *N*^6^-methyladenosine (m^6^A) remains the most thoroughly investigated RNA modification in oncology, governing various biological processes, including cancer metabolism and immunology.^8,9^ However, the in vivo biological functions of m^1^A in tumor immunity and cancer immunotherapy have been largely unexplored.

Oncolytic viruses (OVs) are emerging as a promising cancer immunotherapy because they can convert immunologically “cold” tumors lacking T cell infiltration into “hot” tumors with substantial cytotoxic T cell infiltration.^10^ OVs preferentially lyse cancer cells without harming normal cells, conferring potent antitumor efficacy with limited toxicity.^10^ Beyond direct tumor cell lysis, OVs potently stimulate the host immune system by promoting infiltration of activated natural killer (NK) cells and T cells.^11^ However, a major limitation of OV monotherapy is that such immunostimulation is transient, as the initial T cell influx is followed by an inhibitory tumor microenvironment (TME) with upregulated programmed death-ligand 1 (PD-L1) expression.^12,13^ PD-L1 is induced by cytokines like interferon-gamma (IFN-γ) secreted by activated T cells, representing an adaptive immune resistance mechanism.^12,13^ IFN-γ produced following OV-mediated immunostimulation potently upregulates tumoral PD-L1.^12,13^ This ligand then exhausts PD-1^+^ tumor-infiltrating lymphocytes, limiting virotherapy and representing a target for combination strategies.^12,13^ Selectively inhibiting PD-L1 synthesis after OV treatment may thus sustain OV-induced antitumor immunity by rescuing T cells from exhaustion.

The oncogene *MYC*, also known as *c-MYC*, belongs to a family of genes whose protein products are frequently overactivated in human cancers.^14^ As a transcription factor regulating thousands of genes directly or indirectly, MYC is a master controller of multiple biological programs.^15,16^ In addition to its cell-intrinsic effects, MYC also influences host immunity and the tumor microenvironment.^17^ Numerous studies have shown that MYC overexpression can cause cancer. For example, transgenic MYC overexpression in mice was sufficient to cause B-cell lymphoma.^18^ Additional research confirmed MYC’s ability to induce various cancer types in humans.^19,20^ MYC-induced tumors regressed rapidly and dramatically when MYC was inactivated in mouse models, even without affecting normal endogenous MYC.^21,22^ This phenomenon, called oncogene addiction, suggests targeting MYC could effectively treat some human cancers. MYC regulation of PD-L1 expression directly participates in MYC-driven tumorigenesis initiation and continuation.^17^ MYC overexpression may constitute a general mechanism whereby tumor cells upregulate immune checkpoint regulator expression, thereby circumventing immune surveillance.^17^ MYC inactivation has been proposed to reinstate antitumor immune responses.^23,24^ However, no drug has yet demonstrated effective therapeutic targeting of MYC or its pathway, although promising candidates are in development.^25^

In this study, we found that m^1^A modification levels were significantly increased in both oral dysplasia and HNSCC. Mechanistically, TRMT61A regulated HNSCC progression and immunosuppression by promoting MYC oncoprotein synthesis and inducing PD-L1 expression. Our results further suggest that TRMT61A activates the MYC pathway by enhancing MYC translation instead of transcription, indicating epigenetic control of this oncogene. Additionally, m^1^A inhibition augmented the antitumor immune response elicited by oncolytic herpes simplex virus (oHSV). Our data comprehensively delineate TRMT61A-mediated regulation of the MYC-PD-L1 signaling axis at the translational level, unveiling a novel mechanism governing cancer progression and immunotherapy response.

## Results

### RNA m^1^A modification levels are upregulated during HNSCC progression

**Fig. 1:**
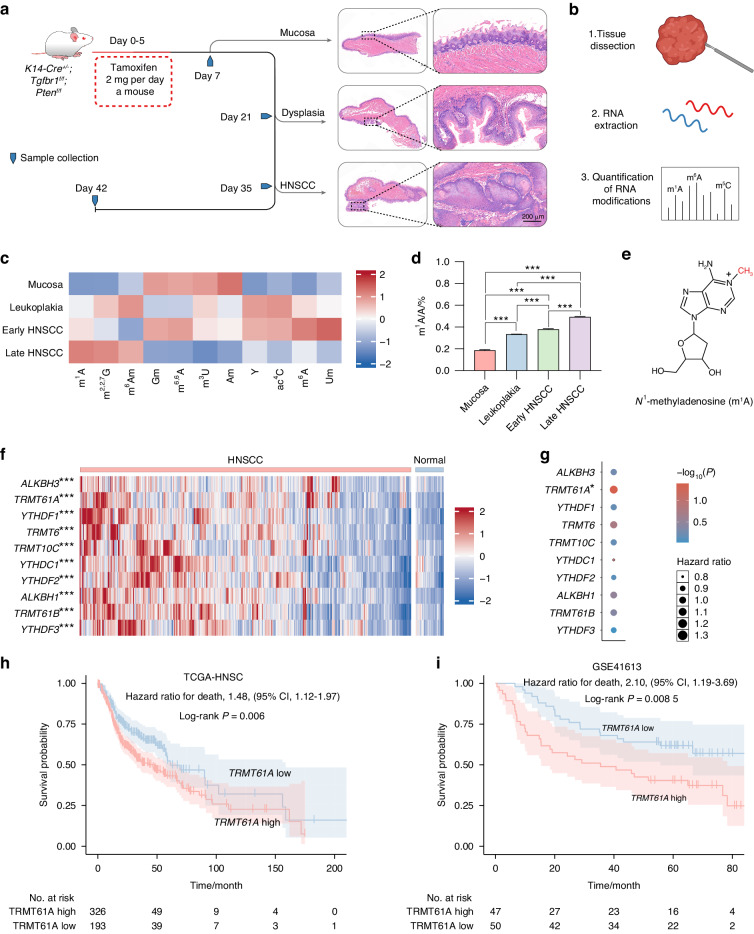
RNA m^1^A modification is activated during HNSCC initiation and progression. **a**, **b** Schematic depicting LC-MS/MS assays. **c** The levels of various RNA modifications in total RNAs, as detected by LC-MS/MS (*n* = 3). **d** The internal m^1^A/A levels in total RNAs, as detected by LC-MS/MS (*n* = 3). **e** The chemical structure of m^1^A methylation. **f** Expression of genes related to m^1^A modification in the TCGA-HNSC dataset. **g** Cox regression of m^1^A-associated genes in the TCGA-HNSC dataset. **h**, **i** Kaplan‒Meier survival analysis of *TRMT61A* in the **h** TCGA-HNSC dataset and **i** GSE41613 datasets at the best cutoff values. One-way ANOVA followed by Tukey’s multiple comparisons tests (**d**); Mann–Whitney test (**f**); Cox regression (**g**–**i**); log-rank test (**h**, **i**). *P < 0.05; **P < 0.01; ***P < 0.001

Histologically, progression from cellular atypia to varying degrees of dysplasia can ultimately lead to invasive HNSCC.^2^ To examine dynamic changes in the RNA modification landscape during HNSCC initiation and progression, we utilized *Tgfbr1* and *Pten* conditional knockout (2cKO) mice, a model that develops dysplasia progressing to HNSCC upon tamoxifen administration (Figs. 1a, b and S1a).^26^ Liquid chromatography-tandem mass spectrometry (LC-MS/MS) screening of total RNA identified over ten RNA modifications, among which m^1^A levels steadily increased during cancer initiation and progression (Fig. 1c–e). We, therefore, hypothesized elevated m^1^A modification plays an important role in malignant transformation and immune evasion in HNSCC. To validate the LC-MS/MS screening results, we assessed m^1^A levels in tumors and peritumoral tissues by immunohistochemistry (IHC). Consistent with LC-MS/MS, m^1^A levels were significantly higher in tumor beds compared to peritumoral regions (Fig. S1b). m^1^A methylation is one of the most prevalent and highly conserved modifications in both eukaryotic and prokaryotic RNAs, suggesting a critical biological role. Despite unclear m^1^A functions in RNAs, these results suggested regulatory effects of m^1^A in HNSCC initiation and progression. m^1^A levels, modification patterns, and regulator expression might closely associate with tumorigenesis.

### TRMT61A is the key m^1^A regulator gene in HNSCC

Similar to dynamic m^6^A modification, m^1^A is installed by methyltransferases called “writers” (including TRMT6, TRMT61A, TRMT10C, and TRMT61B), removed by demethylases called “erasers” (including ALKBH3 and ALKBH1), and recognized by m^1^A-binding proteins called “readers” (including YTHDF1, YTHDF2, YTHDF3, and YTHDC1). To identify which m^1^A regulator contributes most to HNSCC progression, we examined the expression of m^1^A writers, erasers, and readers in the TCGA-HNSC cohort. mRNA levels of m^1^A regulators were significantly elevated in tumors compared to normal tissues, indicating activation of the m^1^A machinery in cancer (Fig. 1f). Among known m^1^A writers, TRMT61A primarily modifies cytosolic tRNAs, while TRMT61B and TRMT10C mediate m^1^A modification on mitochondrial RNA.^27,28^ TRMT61A contains a methyl donor (*S*-adenosyl-l-methionine, SAM) binding pocket and functions as the catalytic subunit. It forms a functional heterotetramer complex with TRMT6, which lacks the SAM binding motif but is essential for tRNA-binding.^6^ We found *TRMT61A* mRNA was elevated in both paired (Fig. S1c) and unpaired (Fig. S1d) tumor samples compared to normal samples. Further analysis revealed upregulated *TRMT61A* mRNA in various human cancers, including liver, breast, and colon (Fig. S2). Cox regression showed high *TRMT61A* expression conferred the highest hazard ratio (1.011–1.719, *P* = 0.0416) among these genes, while other m^1^A regulators were not significant (Fig. 1g). Interestingly, gene set enrichment analysis (GSEA) revealed marked activation of cell proliferation pathways and suppression of immunological pathways in *TRMT61A*-high tumors (Fig. S3a). Moreover, high *TRMT61A* predicted poor prognosis in both TCGA-HNSC and GSE41613 cohorts (Figs. 1h, i and S3b, c). Together, these findings identified TRMT61A as a key m^1^A regulator in cancer, warranting further study.

### TRMT61A expression is associated with HNSCC progression and poor prognosis

**Fig. 2:**
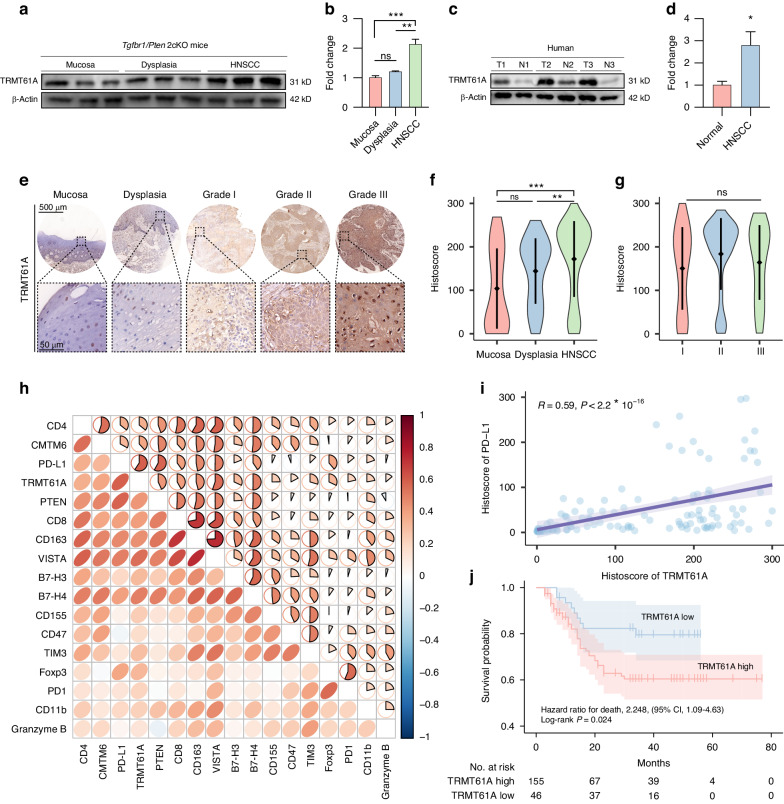
TRMT61A is upregulated in HNSCC tissues and predicts a poor prognosis. **a**, **b** Western blot detection (**a**) and normalized levels (**b**) of TRMT61A expression in normal oral mucosa, dysplasia, and HNSCC tissues of *Tgfbr1/Pten* 2cKO mice. **c**, **d** Western blot detection (**c**) and normalized levels (**d**) of TRMT61A expression in tumor and peritumoral normal tissues of HNSCC patients. **e** Representative IHC staining results of TRMT61A in normal mucosa, dysplasia, and HNSCC samples with different pathological grades. **f** Normalized TRMT61A expression in HNSCC (*n* = 210) compared with dysplasia (*n* = 69) and oral mucosa (*n* = 42). **g** Quantification of the TRMT61A histoscores among pathological grades. **h** Correlation of TRMT61A with other cancer-associated proteins in the HNSCC tissue microarray database. **i** Correlation of TRMT61A with PD-L1 in the HNSCC tissue microarray database. **j** Kaplan‒Meier survival analysis of TRMT61A in the HNSCC tissue microarray database at the best cutoff value. Data were mean with s.e.m. One-way ANOVA followed by Tukey’s multiple comparisons tests (**b**); Kruskal–Wallis test followed by Dunn’s multiple comparisons tests (**f**); Kruskal–Wallis test (**g**); two-tailed paired Student’s *t*-test (**d**); Spearman’s correlation (**h**, **i**); Cox proportional-hazards model and log-rank test (**j**). *P < 0.05; **P < 0.01; ***P < 0.001; ns represents no significance

In *Tgfbr1/Pten* 2cKO mice, Western blot showed spontaneous neoplastic transformation of oral mucosa was accompanied by increased TRMT61A protein levels (Fig. 2a, b). In HNSCC patients, TRMT61A expression was significantly higher in tumors versus peritumoral normal tissues (Fig. 2c, d). We investigated TRMT61A expression by IHC in HNSCC tissue microarrays comprising 42 oral mucosa, 69 dysplasia, and 210 HNSCC samples. Aligning with bioinformatic and Western blot data, IHC revealed higher TRMT61A expression in HNSCC versus dysplasia and normal mucosa (Fig. 2e, f). Further analysis demonstrated positive correlations between TRMT61A and lymph node metastasis, but not tumor size and pathologic grade (Figs. 2g and S4a, c–e). Moreover, metastatic lymph node tissue exhibited higher IHC scores than primary tumors (Fig. S4a–b). TRMT61A did not associate with other clinical parameters including age, smoking, alcohol, or radiotherapy (Table S4). Importantly, TRMT61A positively correlated with PD-L1 (Fig. 2h, i). High TRMT61A predicted poor clinical outcomes in HNSCC patients (Fig. 2j) and was an independent risk factor for overall survival (Fig. S4f, g).

To examine TRMT61A expression in the HNSCC tumor microenvironment (TME), we analyzed a single-cell sequencing dataset of 19 primary HNSCC tumors.^29^ Cell type annotation identified 9 populations, including cancer cells, fibroblasts, and various immune cells (Fig. S5a). *TRMT61A* was universally expressed across all cell types in HNSCC, with the highest levels in malignant cells and fibroblasts (Fig. S5b, c). Confocal microscopy demonstrated cytoplasmic localization of TRMT61A in CAL 27 and WSU-HN6 cells (Fig. S4h, i).

### TRMT61A knockdown reduces the activity of the MYC pathway

**Fig. 3:**
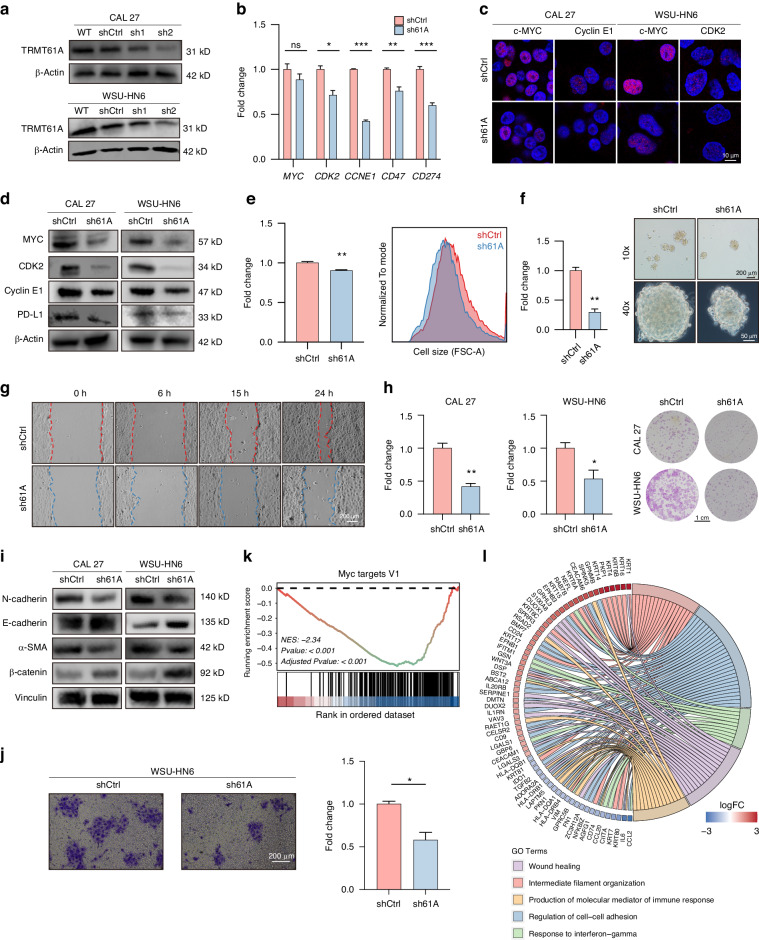
TRMT61A promotes cancer invasion and stemness. **a** Knockdown of TRMT61A by shTRMT61A-2 in WSU-HN6 and CAL 27 cells were confirmed by Western blot. **b** mRNA levels of shCtrl/sh61A CAL 27 cells were quantified by qPCR (*n* = 3). **c**, **d** Protein levels of shCtrl/sh61A WSU-HN6 and CAL 27 cells were quantified by confocal microscopy (**c**) and Western blot (**d**). **e** Cell sizes of shCtrl/sh61A WSU-HN6 cells quantified by FSC-A (*n* = 3). Representative flow cytometry histograms are shown to the right. **f** Sphere formation ability of shCtrl/h61A CAL 27 cells (*n* = 3). Representative flow cytometry histograms are shown to the right. **g** Representative images from in vitro wound healing assays of CAL 27 cells. **h** Colony-forming efficacy of CAL 27 cells (left, *n* = 3) and WSU-HN6 cells (middle, *n* = 3). Representative pictures are shown to the right. **i** Protein levels of epithelial-mesenchymal transition indicators were quantified by Western blot. **j** Representative images of cell invasion assay (left) and quantitative analysis (right). **k**, **l** Analysis of high-throughput sequencing results of sh61A CAL 27 cells against shCtrl CAL 27 cells by a GSEA (**k**; *n* = 3) and a GO enrichment analysis (**l**; *n* = 3). Data were mean with s.e.m. Two-tailed unpaired Student’s *t*-test (**b**, **e**, **f**, **h**, **j**). *P < 0.05; **P < 0.01; ***P < 0.001; ns represents no significance

We screened two lentivirus-mediated TRMT61A shRNAs and selected shTRMT61A-2 (hereafter sh61A) based on superior knockdown efficiency at both mRNA and protein levels (Fig. 3a, S6a). Given previous studies suggest that TRMT61A protein promotes the dramatic transition of quiescent T cells into proliferative T cells^7^ and MYC is the core regulator of T cell activation,^8^ we hypothesize that the MYC pathway is affected upon TRMT61A knockdown. At the mRNA level, known MYC targets like *CCNE1*, *CDK2*, *CD47*, and *CD274* were downregulated in sh61A cells (Figs. 3b, S6b). Interestingly, while *MYC* mRNA was unchanged, MYC protein was reduced in sh61A cells (Fig. 3c, d). Given the decrease in MYC protein, we examined MYC-regulated targets, including CDK2, cyclin E1 (encoded by *CCNE1*), and PD-L1 (encoded by *CD274*). As expected, MYC downregulation decreased cyclin E1, which regulates S phase (Fig. 3c, d and S6c). Among non-canonical targets,^17^ PD-L1 showed reduced mRNA and protein levels (Figs. 3b, d and S6b). Aligning with a previous report,^30^ MYC downregulation decreased cell size (Figs. 3e and S6f). As MYC governs stemness and epithelial-mesenchymal transition, we then examined these phenotypes in shCtrl and sh61A cells. sh61A cells exhibited over 50% lower sphere formation, indicating reduced clonogenicity (Figs. 3f and S6d). Wound healing assays revealed weaker migration and invasion in sh61A cells (Figs. 3g and S6e). Colony formation assays also showed diminished self-renewal upon TRMT61A knockdown (Fig. 3h). Consistent with the upregulated expression of TRMT61A in tumors with higher N levels and lymph metastasis (Fig. S4a–c), Western blot revealed a decrease in the expression of mesenchymal markers, N-cadherin and α-SMA, along with an increase in the expression of the epithelial markers E-cadherin and β-catenin in sh61A cells (Fig. 3i). Furthermore, the cell invasion assay demonstrated a notable reduction in the invasion capability of WSU-HN6 cells following TRMT61A knockdown (Fig. 3j). Taken together, these results provide evidence supporting the role of TRMT61A in promoting cancer stemness and epithelial-mesenchymal transition.

Cancer requires multiple genetic events and the acquisition of hallmarks of cancer.^31^ Importantly, MYC activation can contribute to many hallmarks, including proliferation, self-renewal, survival, genomic instability, metabolism, invasiveness, angiogenesis, and immune evasion.^25^ High-throughput sequencing of shCtrl and sh61A HNSCC cells revealed differentially expressed genes (Figs. S7a, S8a). Gene ontology (GO) enrichment showed involvement of differentially expressed genes in inflammatory and immune responses and cell adhesion (Fig. 3l). GSEA demonstrated marked suppression of cell metabolism and proliferation pathways, including MYC targets, cholesterol homeostasis, mTORC1 signaling, Ras signaling, reactive oxygen species, and oxidative phosphorylation in sh61A cells (Figs. 3k and S7b–d, S8b–h). Thus, TRMT6A has crucial effects in transformed cells and also enables evolving tumors to proliferate and evade the immune response.

### TRMT61A promotes MYC translation but not transcription

Given the unchanged *MYC* mRNA levels and the significant decrease in MYC protein levels, we hypothesize that TRMT61A might promote *MYC* mRNA translation but not transcription. Translational regulation of protein synthesis occurs at initiation, elongation, and termination. tRNA decoding has a fundamental role across these steps. The human genome contains over 400 tRNA genes, with over 200 typically expressed per cell.^32^ tRNAs were long thought to affect translation via structures and mRNA codon interactions. However, their regulation closely relates to diverse tRNA chemical modifications like m^1^A.^7,33^ m^1^A deposition in tRNA is highly conserved and dependent on secondary structure, occurring across life. Cytosolic m^1^A sites share a GUUCNANNC sequence (A = m^1^A) within a hairpin, identical to tRNA T-loops.^27^

**Fig. 4:**
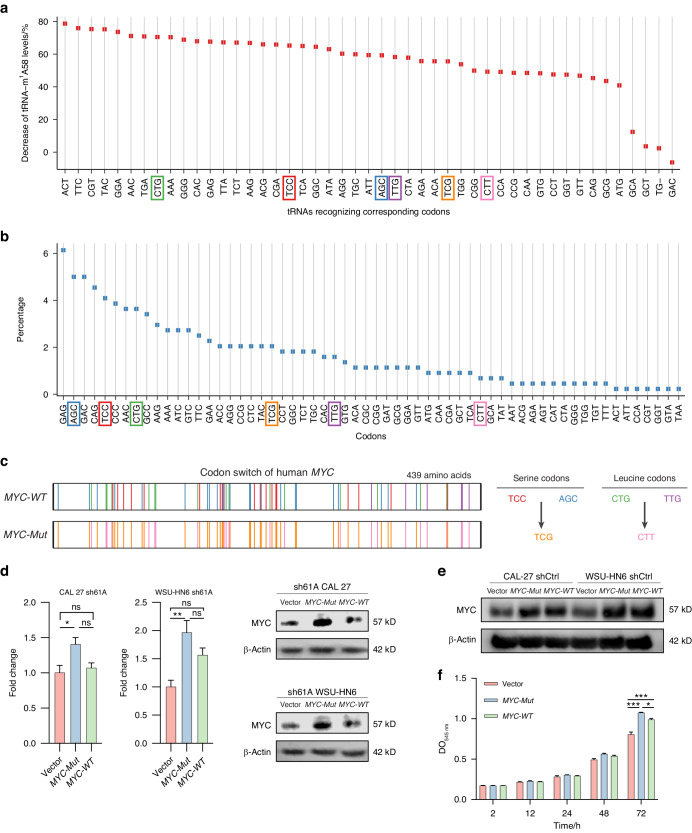
TRMT61A promotes the translation of human *MYC* mRNA. **a** The decrease in the magnitude of the tRNA-m^1^A58 level in each tRNA after TRMT61A deletion. **b** The codon frequency of human *MYC* mRNA. **c** Schematic diagram of the human *MYC* codon-switch assay. **d** Expression of *MYC-WT* and *MYC-Mutant (MYC-Mut)* in sh61A CAL 27 and WSU-HN6 cells. Protein levels of MYC were quantified by immunoblotting (*n* = 4). Representative Western blot results are shown to the right. **e** Expression of *MYC-WT* and *MYC-Mut* in shCtrl CAL 27 and WSU-HN6 cells confirmed by Western blot. **f** Proliferation of sh61A CAL 27 cells transinfected with vector, *MYC-Mut*, and *MYC-WT* detected by SRB assay. Data were mean with s.e.m. Two-tailed unpaired Student’s *t*-test (**d**). One-way ANOVA followed by Tukey’s multiple comparisons tests (**f**). *P < 0.05; **P < 0.01; ***P < 0.001; ns represents no significance

Considering that m^1^A modification is typically found at position 58 of a tRNA, we first reanalyzed the published tRNA-m^1^A-sequence data to demonstrate how TRMT61A knockout leads to the decrease in tRNA m^1^A58 modification levels on different kinds of tRNAs.^7^ Most tRNAs showed varying degrees of reduction in m^1^A58 levels upon TRMT61A knockout, and the most affected tRNAs are the ones decoding serine (TCC and AGC) and leucine (CTG and TTG) (Fig. 4a). Then we compared these tRNAs with codons of the human *MYC* mRNA. Interestingly, TCC, AGC, CTG, and TTG were also among the codons most frequently used by human *MYC* mRNA (Fig. 4b). To further examine the mechanism of *MYC* mRNA translation enhanced by TRMT61A, we redesigned the human *MYC* cDNA by replacing the codons corresponding to the tRNAs most affected by TRMT61A deletion with synonymous codons corresponding to tRNAs least affected by TRMT61A deletion (Fig. 4c). In short, TCC/AGC encoding serine and TTG/CTG encoding leucine were replaced by TCG and CTT, respectively (Fig. 4c). As expected, transfection of *MYC-Mutant* (*MYC-Mut*) plasmid in sh61A CAL 27 and WSU-HN6 cells was enough to significantly upregulate the expression of MYC in two sh61A cells in vitro (Fig. 4d). In sh61A CAL 27 cells, *MYC-WT* plasmid was not able to rescue the defective MYC expression. Although *MYC-WT* plasmids were able to slightly upregulate MYC expression in sh61A WSU-HN6 cells, *MYC-Mut* plasmids rescue the defective MYC expression more efficiently than *MYC-WT* plasmids (Fig. 4d). In shCtrl cells, both *MYC-WT* and *MYC-Mut* plasmids efficiently mediated MYC protein expression, reaching similar protein levels (Fig. 4e), indicating that the translation efficiency is similar for TCC/AGC vs. TCG and CTG/TTG vs. CTT in the presence of TRMT61A. Furthermore, both *MYC-WT* and *MYC-Mut* plasmids increase the proliferation of sh61A CAL 27 cells in vitro, demonstrating that MYC synthesized from *MYC-Mut* plasmids are fully functional (Fig. 4f). Together, these results of this codon-switch assay confirm that tRNA-m^1^A58 modification mediated by TRMT61A directly regulates translation of human *MYC* mRNA via codon decoding.

### TRMT61A promotes PD-L1 expression upon inflammation

**Fig. 5:**
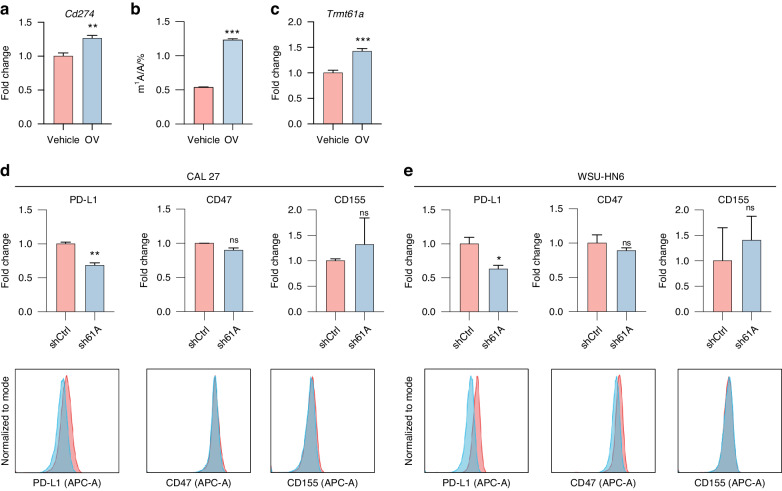
TRMT61A promotes PD-L1 expression upon inflammation. **a** mRNA levels of *Cd274* were quantified by qPCR (*n* = 6 biological replicates per group). **b** The internal m^1^A/A levels in total RNAs of tumors injected with vehicle or OV, as detected by LC-MS/MS (*n* = 3). **c** mRNA levels of *Trmt61a* were quantified by qPCR (*n* = 6 biological replicates per group). **d**, **e** Protein levels of PD-L1, CD155, and CD47 were detected by flow cytometry in shCtrl/sh61A CAL 27 (**d**) and WSU-HN6 (**e**) cells 24 h post 25 ng/mL IFNγ treatment. Representative flow cytometry results are shown at the bottom. Data were mean with s.e.m.*P < 0.05; **P < 0.01; ***P < 0.001; ns represents no significance by two-tailed unpaired Welch’s *t*-test

Previous studies have shown that oncolytic virus (OV) injection induces IFNγ expression, which upregulates PD-L1 expression in cancer cells and leads to immune evasion and OV monotherapy failure.^12^ In *Tgfbr1/Pten* 2cKO mice, two OV doses significantly upregulated mRNA levels of both PD-L1 and TRMT61A (Fig. 5a, c). Interestingly, OV-treated tumors displayed elevated m^1^A modification levels compared to vehicle-injected tumors (Fig. 5b). Since MYC positively regulates PD-L1 expression as a transcription factor, we hypothesize TRMT61A may play a role in OV therapy cancer immune evasion by promoting reactive PD-L1 expression through enabling efficient MYC synthesis. Indeed, upon IFNγ treatment, sh61A CAL 27 and WSU-HN6 cells showed significantly reduced PD-L1 expression compared to shCtrl counterparts, while other immune checkpoint molecules like CD47 and CD155 remained unchanged (Fig. 5d, e). Given MYC also transcriptionally regulates CD47,^17^ we speculate negative feedback loops evolved in sh61A WSU-HN6 cells to impact CD47. Together, these data indicate PD-L1 expression increases with inflammation and TRMT61A at least partially contributes.

**Fig. 6:**
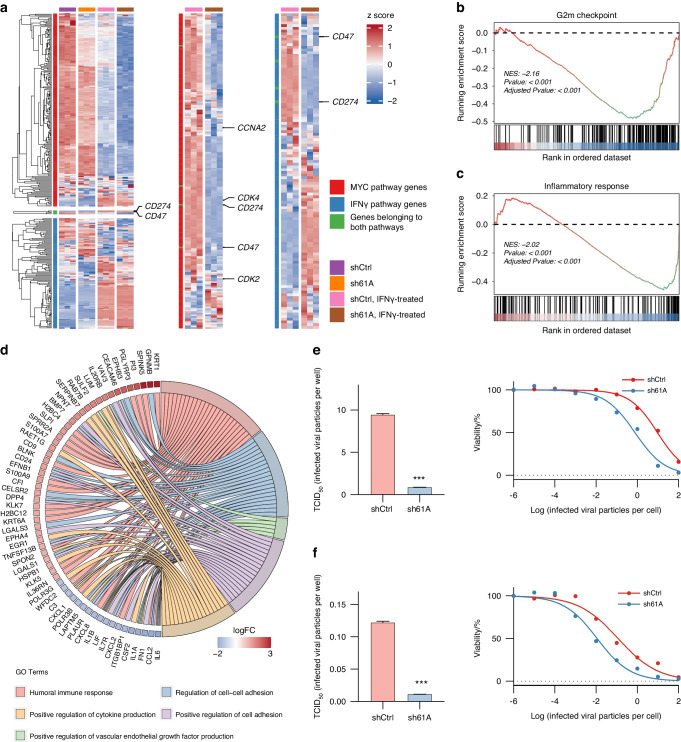
TRMT61A confers resistance against oHSV. **a** CAL 27 expression of genes belonging to MYC target genes or the IFNγ pathway in different groups was identified according to the RNA-seq results. **b**, **d** Analysis of high-throughput sequencing results of IFNγ-treated, sh61A CAL 27 cells against IFNγ-treated, CAL 27 shCtrl cells by a GSEA (**b**, **c**; *n* = 3 biological replicates per group), a GO enrichment analysis (**d**; *n* = 3 biological replicates per group). **e**, **f** Results of the cytotoxicity assay for shCtrl/sh61A CAL 27 (**e**) or WSU-HN6 (**f**) cells upon oHSV infection following IFNγ pretreatment at 25 ng/mL for 24 h (*n* = 3 biological replicates per group). Cell viability was measured three days post infection. Representative log-transformed dose-response curves are shown to the right. Data were mean with s.e.m. Two-tailed unpaired Welch’s *t*-test (**e**, **f**). ***P < 0.001

Next, we sought to explore how TRMT61A regulates the landscape of inflammation using high-throughput sequencing techniques. OV injection activates a spectrum of immune responses, among which IFNγ signaling is significantly upregulated in both injected lesions and noninjected lesions.^11^ At the same time, it has been reported that IFN-γ are responsible for the upregulation of PD-L1 on tumors.^34^ Thus, IFNγ-treated cancer cells are chosen as an in vitro model simulating an inflamed TME post-OV treatment. Upon IFNγ treatment, the MYC target genes and the IFNγ pathway genes could be classified into three subsets based on mRNA expression profiles, suggesting a regulatory program during inflammation (Fig. 6a). As observed in WSU-HN6 cells, the MYC target genes are generally downregulated in sh61A CAL 27 cells in comparison to shCtrl CAL 27 cells, with or without IFNγ treatment (Fig. 6a). Interestingly, IFNγ treatment also inhibits the transcription of most MYC target genes, indicating that proliferation is inhibited during inflammation (Fig. 6a). Reculstering of MYC target genes in IFNγ treated cells shows that genes related to cell cycle regulation, such as *CDK4*, *CCNA2*, and *CKD2*, are downregulated during inflammation (Fig. 6a). As excepted, the IFNγ pathway is activated upon IFN-γ treatment, leading to upregulation of immune checkpoint molecules such and *CD274* and *CD47* (Fig. 6a). Reculstering of IFNγ pathway genes showed a shifted expression profile in the IFNγ pathway in sh61A CAL 27 cells, with pronounced downregulation of *CD274* and *CD47* (Fig. 6a), reinforcing the conclusion that TRMT61A is critical to the reactive upregulation of PD-L1 on cancer cells during inflammation.

GSEA of differentially expressed genes in IFNγ-treated sh61A versus shCtrl CAL 27 cells showed marked suppression of cell cycle and inflammation pathways, including G2M checkpoints, inflammation, MYC targets, E2F targets, and KRAS signaling (Figs. 6b, c and S9a-c). GO analysis revealed many were involved in immune responses like cytokine production and cell adhesion (Fig. 6d). To gain a general understanding of the impact of TRMT61A on OVs, we examined the cytotoxicity of oHSV against shCtrl and sh61A HNSCC cell lines. In both sh61A cell lines, cytotoxic assays demonstrated TRMT61A knockdown increased oHSV sensitivity, leading to a more than 90% percent decrease in 50% tissue culture infectious dose (TCID_50_) following oHSV treatment in comparison to their shCtrl counterparts (Fig. 6e, f).

### Inhibition of TRMT61A activity elevates oncolytic viruses-induced antitumor immunity

**Fig. 7:**
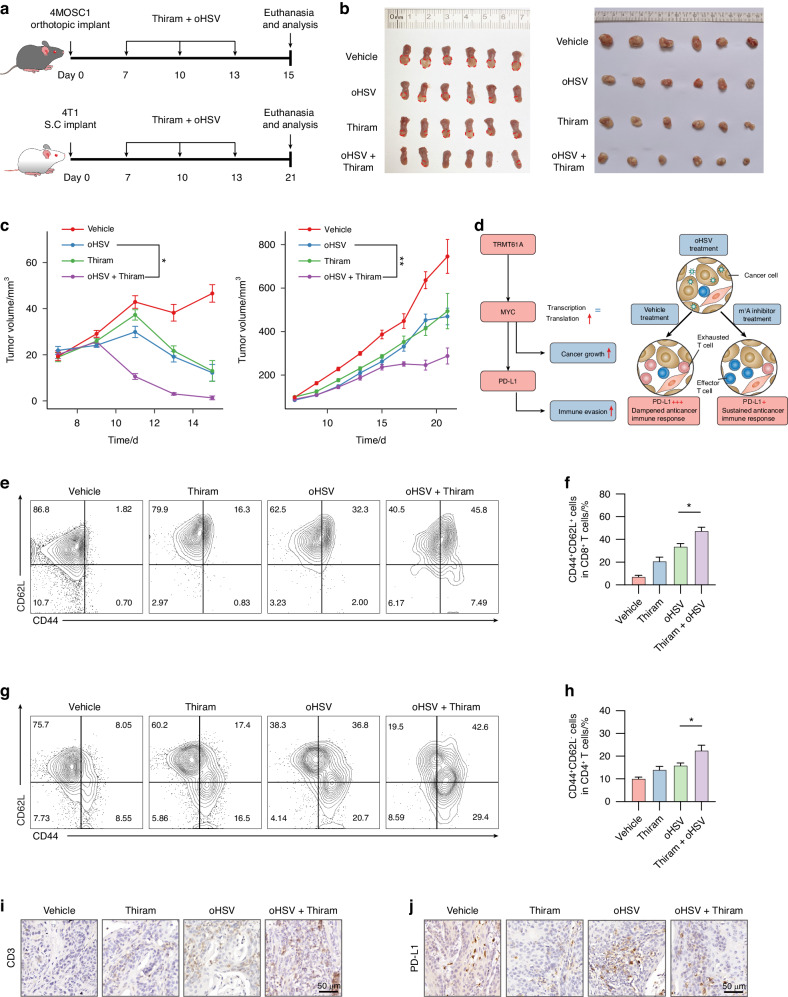
Thiram enhanced oHSV efficacy in two immunocompetent allograft mouse models. **a** Schematic depicting the study design for 4MOSC1 (up panel) and 4T1 (down panel) tumor inoculation and treatment with oHSV and thiram. **b** Macroscopic appearance of tumors from 4MOSC1 (left panel) and 4T1 (right panel) at the end of the experiment. **c** Tumor-growth curve of 4MOSC1 (left panel) and 4T1 tumors (right panel). **d** Proposed mechanism of combining oHSV and m^1^A inhibition. **e**, **f** Representative flow cytometric analysis images (**e**) and quantification (**f**) of CD44 and CD62L expression in the CD8^+^ T cells from TDLNs of 4MOSC1 tumors. **g**, **h** Representative flow cytometric analysis images (**g**) and quantification (**h**) of CD44 and CD62L expression in the CD4^+^ T cells from TDLNs of 4MOSC1 tumors. **i**, **j** Representative IHC images of CD3 epsilon (**i**) and PD-L1 (**j**) of 4MOSC1 tumors. Two-tailed unpaired Student’s t-test (**c**, **f**, **h**). Data were mean with s.e.m (*n* = 6 mice per group). *P < 0.05; **P < 0.01; ***P < 0.001; ns represents no significance

Encouraged by promising in vitro results, we speculated therapies suppressing TRMT61A activity could restore immune responses against cancers during OV therapy. Compared to OV monotherapy, the thiram and oHSV combination therapy showed the most promising antitumor effect (Fig. 7a–c). In the 4MOSC1 syngeneic oral cancer model, combination therapy eradicated tumors in two-thirds of cases by the study end, while no tumors were eradicated with oHSV alone (Fig. 7a–c). Aligning with a previous study reporting thiram as an inhibitor blocking enzymatic activity of the TRMT6/TRMT61A complex,^35^ thiram treatment did not significantly affect TRMT61A protein levels (Fig. S10a), whereas the levels of m^1^A modification were decreased in the tumors of both 4MOSC1 and *Tgfbr1/Pten* 2cKO HNSCC models (Fig. S10b, c).

We speculate in immunocompetent models, TRMT61A inhibition downregulates MYC and PD-L1 simultaneously, dampening inflammation-induced immune inhibition post-oHSV (Fig. 7d). Upon MYC downregulation, loss of “don’t find me” signals enables residual tumor cell destruction and sustained regression. To test this, we examined the post-treatment immune landscape in tumor tissue, tumor-draining lymph nodes (TDLNs), and spleen by flow cytometry. T cell factor 1 (TCF-1), a Wnt pathway transcription factor, maintains CD8^+^ T cell stemness in the tumor microenvironment and during alloimmunity.^36,37^ Combination therapy significantly increased intratumoral TCF-1^+^ T cell fractions versus oHSV alone in a 4T1 breast cancer model (Fig. S11a–c). Beyond TCF-1, CD44 and CD62L classify T cells into central memory (T_cm_), effector (T_eff_), and naïve subsets.^38^ TDLN CD8^+^ T cells showed increased T_cm_ but not T_eff_ with combination treatment (Figs. 7e, f and S1a, b). Interestingly, in TDLN CD4^+^ T cells, T_cm_ numbers showed no significant increase, while T_eff_ significantly increased with combination therapy (Figs. 7g, h and S12a, c). Classically activated M1 macrophages have greater antitumor effects than alternatively activated M2 types.^39^ Combination therapy dramatically elevated intratumoral MHCII^+^F4/80^+^ M1 macrophages versus oHSV alone (Fig. S13a–c). Mature dendritic cells (DCs) expressing CD80/86 provide secondary signals activating T cells, and combination therapy increased CD80^+^CD86^+^ DCs in 4MOSC1 TDLNs (Fig. S14a–c).

To explore the correlation between TRMT61A and PD-L1-mediated immune evasion, we evaluated the death of si*Trmt61a*-transfected 4MOSC1 cancer cells when co-cultured with CD8^+^ cells isolated from mice cured by the thiram and oHSV combination therapy (Fig. S15a–c). These CD8^+^ T cells exhibited substantial cytotoxicity against 4MOSC1 cells, a response that was further enhanced by the introduction of *Trmt61a* siRNA or PD-L1 antibodies (αPD-L1). Interestingly, the simultaneous application of both *Trmt61a* siRNA and αPD-L1 did not yield a significant improvement in cytotoxicity (Fig. S15c), suggesting overlapping pharmacological mechanisms. Additionally, IHC showed increased T cell infiltration with combination therapy, while oHSV-induced PD-L1 upregulation was decreased (Fig. 7i), reinforcing TRMT61A’s critical role in reactive PD-L1 upregulation during oHSV treatment. In *Tgfbr1/Pten* 2cKO mice, thiram treatment also dramatically reduced tumor growth rate (Fig. S16a–d). Although modest, MYC’s effects on PD-L1 expression had dramatic tumor regression consequences, consistent with minor immune regulator influences markedly impacting outcomes.^17^ In summary, the thiram and oHSV combination demonstrated enhanced antitumor efficacy and immune modulation versus oHSV monotherapy.

## Discussion

Choosing an appropriate cancer model is critical for studying RNA modifications during cancer initiation, progression, and response to immunotherapy. HNSCC is a suitable model as it has a well-defined precancerous state^40^ and is superficial, allowing for noninvasive monitoring of cancer progression. Before invasive cancer arises, some patients may present with oral premalignant lesions, including leukoplakia (white patches), erythroplakia (red patches), or dysplastic leukoplakia.^40^ While previous research has examined the role of m^1^A in cancers like hepatocellular carcinoma and bladder cancer, m^1^A modification levels in precancerous tissues remained unclear.^35,41^ Using our *Tgfbr1/Pten* 2cKO mice, we demonstrate elevated RNA m^1^A modification levels in precancerous states. Although evidence indicates a histological progression from cellular atypia to varying degrees of dysplasia ultimately culminating in invasive HNSCC, most patients present with late-stage HNSCC lacking a clinically detectable precursor premalignant lesion.^2^ With the development of swift, low-cost nucleotide modification detection technologies,^42^ RNA m^1^A levels may become a new routine biomarker for cancer prevention and early diagnosis.

Although MYC is a broad transcriptional amplifier utilizing diverse mechanisms, it also displays transcriptional effects based on gene dosage.^43^ The relatively high MYC levels linked to rapid proliferation and tumorigenesis may induce PD-L1 expression. With MYC regulating its gene transcription, PD-L1 may exhibit higher steady-state expression compared to other membrane proteins in tumors. Notably, MYC activation of the PD-L1 gene appears to require greater MYC promoter binding than genes involved in normal cell growth; hence, they may constitute promoters invaded by oncogenic MYC levels and be especially sensitive to MYC withdrawal.^44^ Through suppressing immune surveillance against tumor cells, TRMT61A may impact cancer immunoediting. We propose high TRMT61A expression during tumor evolution increases PD-L1 expression, suppressing adaptive immune responses to promote tumor growth. We speculate that TRMT61A suppression may restore antitumor immune responses in human cancers. Cancers overexpressing TRMT61A may be especially susceptible to thiram treatment.

Currently, management of HNSCC is usually multimodal, involving surgery followed by chemoradiotherapy (CRT) for oral cavity cancers and primary CRT for pharynx and larynx cancers.^2^ The EGFR antibody cetuximab frequently combines with radiation for HPV-negative HNSCC when coexisting conditions impede cytotoxic chemotherapy utilization.^45^ The FDA approved the immune checkpoint inhibitors (ICIs) pembrolizumab and nivolumab for recurrent/metastatic HNSCC treatment, with pembrolizumab also endorsed as initial therapy for unresectable disease.^46–48^ Although ICIs have significantly prolonged the survival of recurrent or metastatic HNSCC, about 80% of advanced-stage patients remain unresponsive to ICIs,^46–48^ possibly due to various mechanisms of resistance to ICIs. An emerging consensus in the field of cancer immunotherapy is that immune “cold” tumors with scarce T cell infiltration show poorer responses to ICIs.^49^ One advantage of OVs is the ability to selectively replicate in and lyse cancer cells, inducing immunogenic cell death and converting an immune “cold” tumor into a “hot” one.^10^ However, virus-induced inflammation may remodel the tumor microenvironment, upregulating PD-L1 expression and dampening OV-induced antitumor immunity, ultimately causing OV therapy failure.^12,13^ Methodologically, two strategies counteract reactive PD-L1 upregulation: inhibition of PD-L1 function or inhibition of de novo PD-L1 synthesis. Most published studies combine PD-L1 antibody blockade with OVs, inhibiting PD-L1 function.^12,13^ An issue with PD-L1 antibodies is theoretical efficacy is limited to membrane and serum PD-L1, with little influence on cytoplasmic and nuclear PD-L1.^50^ In fact, intracellular PD-L1 promotes tumorigenesis and dampens immunotherapy efficacy.^51^ The second strategy, using agents like the thiram in this study, may overcome this limitation by inhibiting PD-L1 protein synthesis, theoretically reducing abundance regardless of location. Given the absence of a specific drug directly designed to interact with MYC, largely due to its non-possession of an enzymatic pocket and inaccessibility to antibodies, thiram represents an alternative strategy for addressing MYC as a target in cancer treatment, focusing on the regulation of *MYC* mRNA translation.^52^ The superior efficacy of *Trmt61a* siRNA compared to αPD-L1 may be attributed to its ability to inhibit the MYC pathway (Fig. S15b, c). Thus, by reducing the production of “undruggable” MYC and inhibiting de novo PD-L1 synthesis, m^1^A inhibitors are promising candidates for overcoming acquired resistance to OV therapy, with distinct advantages over antibody-based PD-L1 immunotherapy.

We demonstrated RNA m^1^A is an essential and druggable cancer modification, especially in HNSCC. Given that m^1^A modifications on mRNAs lead to translational repression and thus are generally avoided by cells,^27^ we emphasized the importance of tRNA m^1^A modification in our study. tRNAs have over 90 modifications, averaging about 13 per tRNA.^53^ These numerous tRNA modifications greatly limit studying any single modification’s function. Open questions remain about whether m^1^A cooperates with other tRNA modifications to shape folding, whether undiscovered m^1^A sites exist in tRNAs, and what the exact m^1^A function is in tRNAs. Additionally, m^1^A occurs in a small number of mRNAs, typically at low stoichiometry. This low mRNA m^1^A stoichiometry has implications for how m^1^A could affect mRNAs. It is commonly believed that m^1^A regulates target expression as a mechanism responding to environmental changes. However, the impact of low stoichiometry m^1^A in mRNAs on target regulation requires consideration. Although TRMT61A modestly impacted PD-L1 expression, tumor repression effects were marked, aligning with studies showing small immune checkpoint changes dramatically influence outcomes.^17,54^ Our results demonstrate that MYC-mediated PD-L1 translation modulation is the primary TRMT61A immunosuppression mechanism. Beyond PD-L1, MYC transcriptionally regulates CD47, though our study showed no significant CD47 fluctuation. Considering that TRMT6/TRMT61A can catalyze m^1^A in mRNAs with tRNA T-loop-like structures, we postulate CD47 expression feedback loops related to mRNA m^1^A modifications.

This study establishes a model elucidating how m^1^A-modified tRNA enables cancer progression and immune evasion through epigenetic translational modulation. Following m^1^A function examination in mouse T cells, we extended translational modulation investigations to human cancers, among the deadliest diseases worldwide. Over the past decade, elucidating the HNSCC molecular genetic landscape has uncovered new opportunities for therapeutic intervention.^55–57^ Current efforts aim to integrate an understanding of HNSCC biology and immunobiology to pinpoint predictive biomarkers enabling the administration of the most effective, least toxic individualized therapies. Given increasing OV attention after the 2015 T-VEC approval, this novel OV resistance alleviation combination strategy might be tested in future clinical trials. Synthesis inhibition could provide advantages over current PD-L1 antibody blockade, including reduced immunosuppressive signaling from intracellular PD-L1. Overall, selectively blocking PD-L1 production after OV treatment may sustain antitumor immunity by preventing the exhaustion of newly infiltrating cytotoxic T cells. Further research into epitranscriptomic regulators like m^1^A provides an exciting avenue for enhancing cancer immunotherapies. Looking forward, tRNA level epigenetic regulation likely plays roles in many biological contexts.

## Materials and methods

Details are provided in Supplementary information.

### Cells and RNA interference

The human head and neck cancer cell line WSU-HN6 was obtained from Ninth People’s Hospital, Shanghai Jiao Tong University School of Medicine (Shanghai, PR China). The murine breast cancer cell line 4T1 (cat. no. CRL-2539) and the human head and neck cancer cell line CAL 27 (cat. no. CRL-2095) was purchased from American Type Culture Collection (Manassas, VA, USA). Complied with a material transfer agreement (SD-2017-202), 4MOSC1 cells were generous gifts from Prof. J. Silvio Gutkind (University of California San Diego, USA) and were cultured in keratinocyte serum-free medium (K-SFM, Gibco-BRL).^58^ WSU-HN6 and CAL 27 cells were cultured in DMEM/high glucose medium (HyClone) supplemented with 10% fetal bovine serum (Gibco) and 1% penicillin/streptomycin (HyClone). 4T1 cells were cultured in RPMI 1640 medium modified (HyClone) supplemented 10% fetal bovine serum (Gibco) and 1% penicillin/streptomycin (HyClone). All cells are regularly examined with a *Mycoplasma* detection kit (Servicebio, cat. no. G1900) to make sure there is no *Mycoplasma* contamination. In this study, cells were cultured for no more than 15 generations in total.

For shRNA-mediated interference, two effective shRNAs against TRMT61A used in this study have been validated previously.^35^ The sequences of the shRNAs used are as follows: ctrl shRNA: 5′-GTTCTCCGAACGTGTCACGT-3′, *TRMT61A* shRNA-1: 5′-GGCACTCAGTTGACCTTAT-3′, *TRMT61A* shRNA-2: 5′-GCATACGAGGAGCTGATCAAG-3′. shRNAs against TRMT61A and control hairpins were cloned into the LV3 vector. High-titer lentivirus stocks were purchased from GenePharma. Production of lentiviral particles and transduction of cancer cells was performed according to protocols from the RNAi consortium (http://www.broadinstitute.org/ rnai/trc). Cells were transfected with lentiviral constructs expressing shRNA or shCtrl as described above at an MOI of 10 for 48 h. Positive cells were selected with puromycin (2 μg/mL) for 7 days. Then, cells were collected for protein and RNA analysis. The sequences of the siRNAs used are as follows: *Trmt61a* sense: 5′-CUGACUUUUGCCACUAAAACC-3′, *Trmt61a* antisense: 5′-UUUUAGUGGCAAAAGUCAGGU-3′; ctrl sense: 5′-UUCUCCGAACGUGUCACGUTT-3′; ctrl antisense: 5′- ACGUGACACGUUCGGAGAATT-3′. The cells were transfected with siRNAs using Lipofectamine^TM^ 3000 (Invitrogen, USA) according to the manufacturer’s instructions.

### RNA-seq and bioinformatic analysis

A total amount of ≥800 ng RNA per sample was used as the starting RNA for library construction. The library construction kit used in library construction is Illumina’s NEBNext Ultra RNA Library Prep Kit. The purified double-stranded cDNA is end-repaired, A-tailed and ligated with sequencing adapters, and AMPure XP beads are used to select cDNA of about 200 bp, PCR amplification and AMPure XP beads purification of PCR products are performed to obtain the library. After the library passes quality control, different libraries are pooled according to effective concentration and target data volume requirements for sequencing, and produce 150 bp paired-end reads. DNBSEQ-T7 platform was adopted by Wuhan GeneRead Biotechnology to sequence the libraries.

### Animal experiments

Animal experimental procedures were approved by the Center for Animal Experiment of Wuhan University (WP20230056, WP20230228) and the Animal Ethics Committee of the School and Hospital of Stomatology of Wuhan University (S07923060N). The mice were maintained under standard conditions according to the institutional guidelines for animal care. Calculation of tumor volume (mm^3^) was applied: width^2^ × length × 0.5.

*Tgfbr1/Pten* 2cKO *mice*.Time inducible tissue-specific *Tgfbr1*/*Pten* 2cKO mice (*K14-Cre*^*ERtam+/−*^; *Tgfbr1*^*flox/flox*^; *Pten*^*flox/flox*^) were kindly provided by Dr. Ashok B. Kulkarni (National Institute of Dental and Craniofacial Research, USA) with material transfer agreement (NIH T-2012-1735) and United States Department of Agriculture certification (VA-12-4122R). The tamoxifen treatment procedure has been previously described.^26^ Briefly, tamoxifen was dissolved in corn oil and administrated orally to the mice for 5 consecutive days (2 mg per day per mouse). Mice were housed in appropriate sterile filter-capped cages, and fed and watered ad libitum. 6- to 8-week-old male and female *Tgfbr1/Pten* 2cKO mice were included in this study. Thiram (1.6 mg/kg) were intraperitoneally injected on days every other day. Tumor growth was detected on days 7, 14, 21, 28, 35, and 42 after treatment.

*Immunocompetent allograft mouse models*. 4T1 (0.7 × 10^6^) cells resuspended in 200 μL of RPMI 1640 medium were subcutaneously injected into the right hind flank of each BALB/c mouse. Seven days post tumor implantation, the mice received intratumoral oHSV (2 × 10^6^ pfu) and/or intraperitoneal thiram (1.6 mg/kg) injection. 4MOSC1 (1 × 10^6^) cells resuspended in 25 μL of DMEM medium were transplanted into the dorsum linguae of each C57Bl/6 mouse. Seven days post tumor implantation, the mice received intratumoral oHSV (0.4 × 10^6^ pfu) and/or intraperitoneal thiram (1.6 mg/kg; Aladdin, cat. no. T111114) injection. The injections were repeated every three days for a total of three times. Tumor growth was measured as indicated.

### Statistical analysis

The data were presented as mean with standard error of the mean (s.e.m.). Unless indicated otherwise, multiple comparisons among more than two groups were performed using one-way ANOVA with Tukey’s multiple comparisons test, and two-group comparisons were analyzed using a two-tailed unpaired Student’s *t*-test. The survival benefit was determined using a log-rank (Mantel–Cox) test. All statistical analyses were conducted using GraphPad Prism software version 9 for Mac OS (GraphPad Software). A significance level of *P* < 0.05 was considered statistically significant for all types of analyses.

### Supplementary information

Supplementary Files

## Data Availability

The RNA-seq data used in this study has been uploaded to the GEO (accession number, GSE247024). Associated data are available upon reasonable request to the corresponding author.
