# Supplementary material for: m1A inhibition fuels oncolytic virus-elicited antitumor immunity via downregulating MYC/PD-L1 signaling
Source: Int J Oral Sci. 2024 May 10;16:36. doi: 10.1038/s41368-024-00304-0 (PMC11087574; doi:10.1038/s41368-024-00304-0)
Supplement: Supplementary file 1 — Supplementary Files [file 41368_2024_304_MOESM1_ESM.pdf]

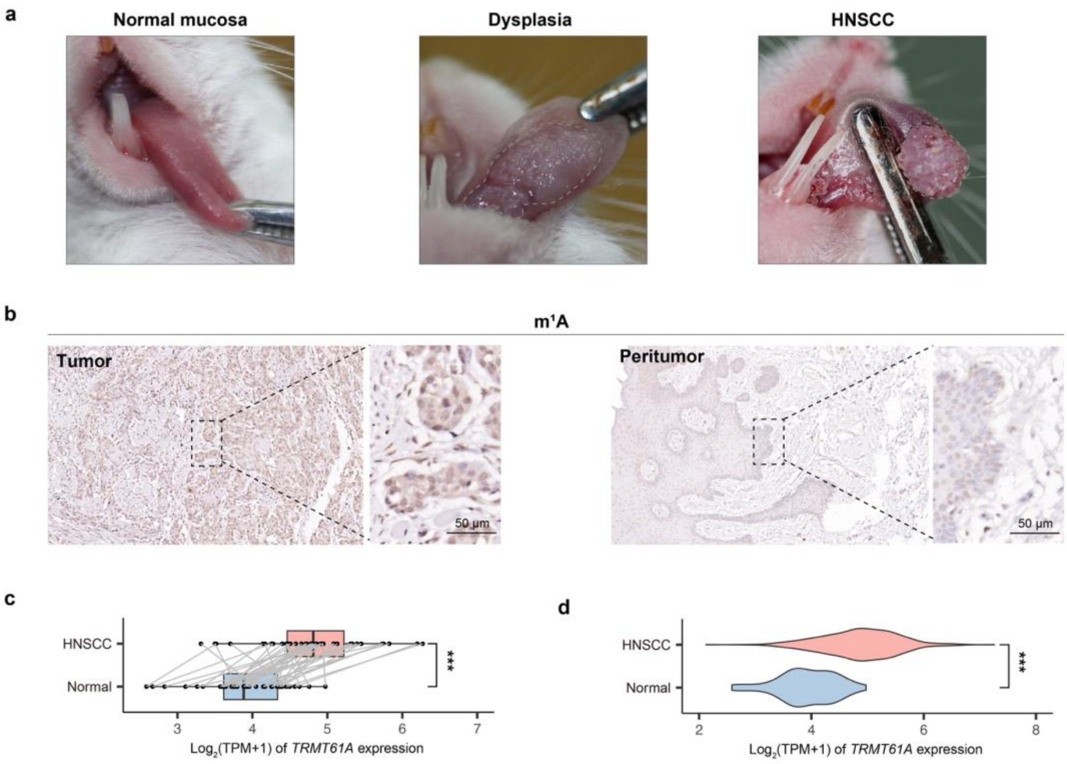

Supplementary figure 1. **a**, Representative images of normal mucosa (left), leukoplakia (middle), HNSCC (right) of *Tgfbr1/Pten* 2cKO mice. **b**, Representative IHC results of m^{1}A in human HNSCC (left panel) and peritumor tissues (right panel). **c-d**, Paired (**c**) and unpaired (**d**) expression of *TRMT61A* in the TCGA-HNSC dataset. Two-tailed t-test (**c, d**). ****P* < 0.001.

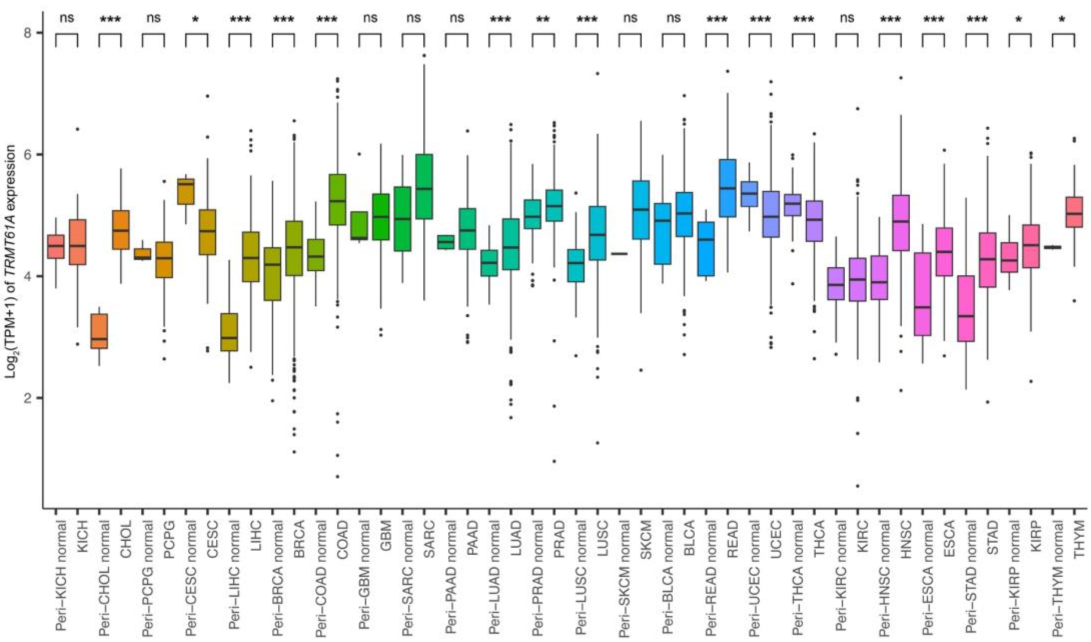

Supplementary figure 2. The expression profile of *TRMT61A* in the TCGA dataset. Please visit the following website for TCGA study abbreviations: https://gdc.cancer.gov/resources-tcga-users/tcga-code-tables/tcga-study-abbreviations. **P* < 0.05; ***P* < 0.01; ****P* < 0.001; ns represents no significance by two-tailed unpaired Student's t-test.

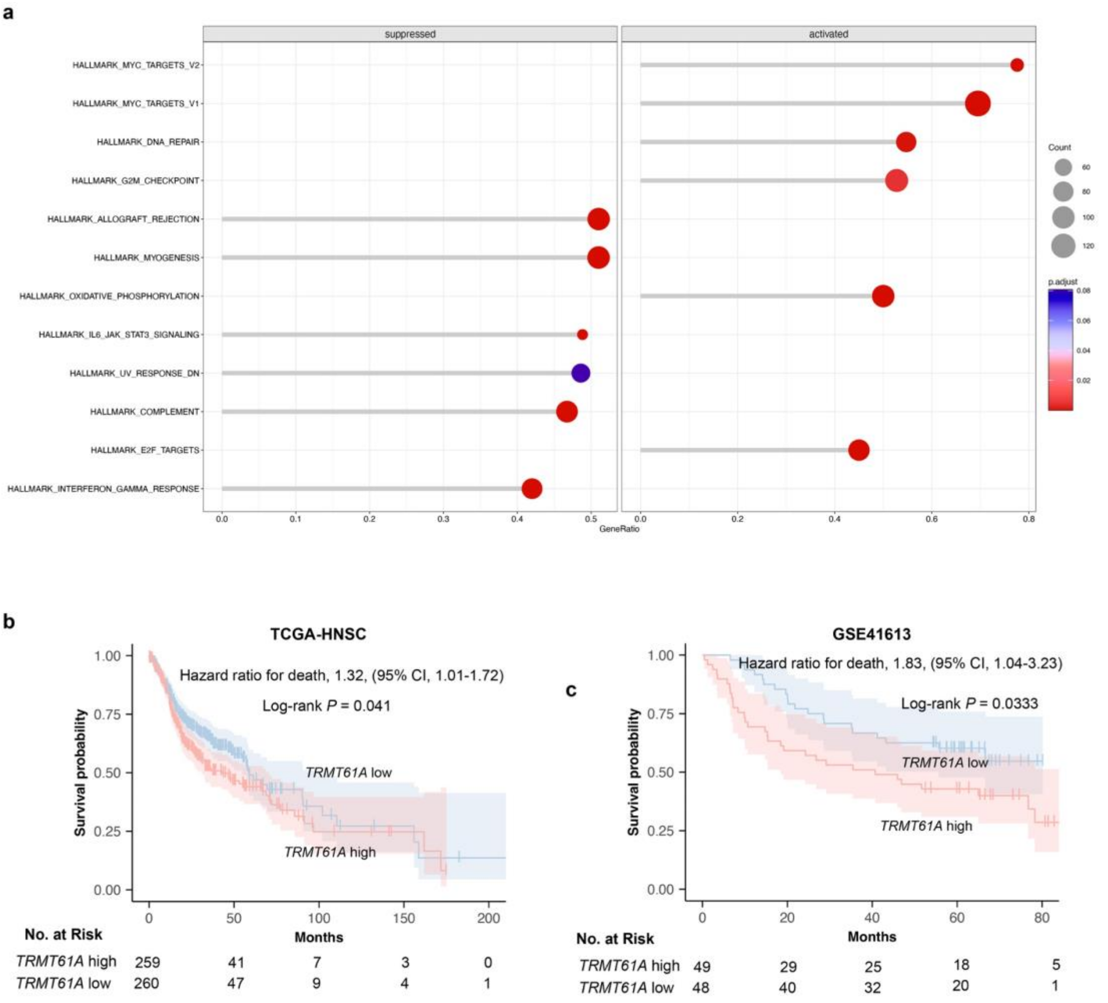

Supplementary figure 3. **a**, Analysis of TCGA-HNSC dataset by comparing tumors expressing high levels of *TRMT61A* (n = 259) against tumors expressing low levels of *TRMT61A* (n = 260) by a GSEA. **b-c**, Kaplan–Meier survival analysis of *TRMT61A* in the (**b**) TCGA-HNSC dataset and (**c**) GSE41613 datasets. Cox proportional-hazards model (**b-c**); log-rank test (**b-c**).

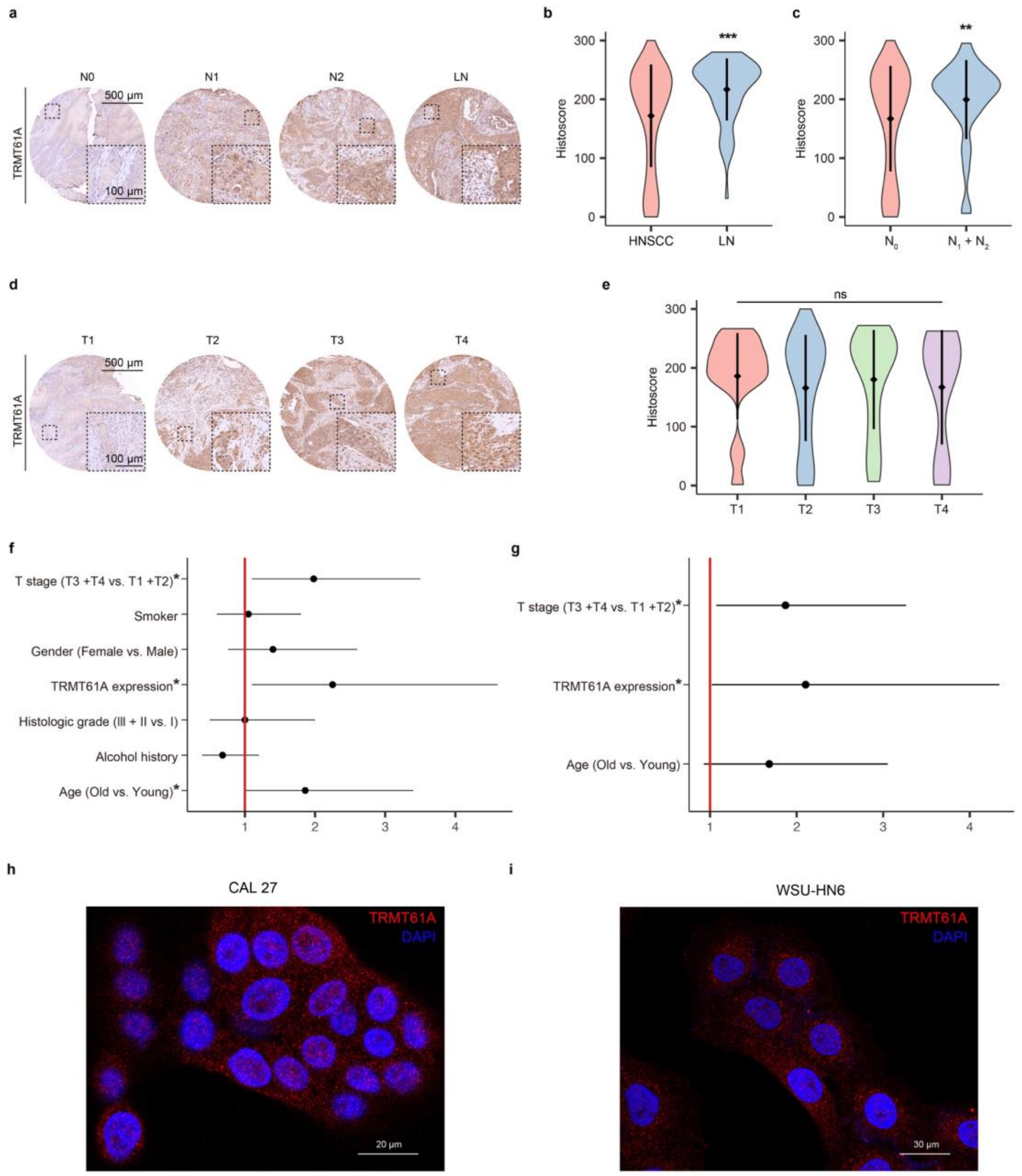

Supplementary figure 4. The expression profile of TRMT61A in the TME. **a**, Representative IHC staining results of TRMT61A in tumor samples of different N stages. **b**, Normalized TRMT61A expression in HNSCC (*n* = 210) compared with lymph nodes (LN, *n* = 54). **c**, Normalized TRMT61A expression in N_0 (*n* = 138) compared with N_1 + N_2 (*n* = 72). **d**, Representative IHC staining results of TRMT61A in tumor samples of different T stages. **e**, Normalized TRMT61A expression in tumor samples of T1, T2, T3 and T4 stages. **f-g**, Forest plots of results from the univariate (**f**) and multivariate (**g**) Cox regression analysis and of TRMT61A in the HNSCC tissue microarray database. **h-i,** Representative confocal images of TRMT61A expression in CAL 27 and

WSU-HN6 cells. Data are mean with s.e.m. Mann-Whitney test (**b-c**); Kruskal-Wallis test (**e**). **P* < 0.05; ***P* < 0.01; ****P* < 0.001; ns represents no significance.

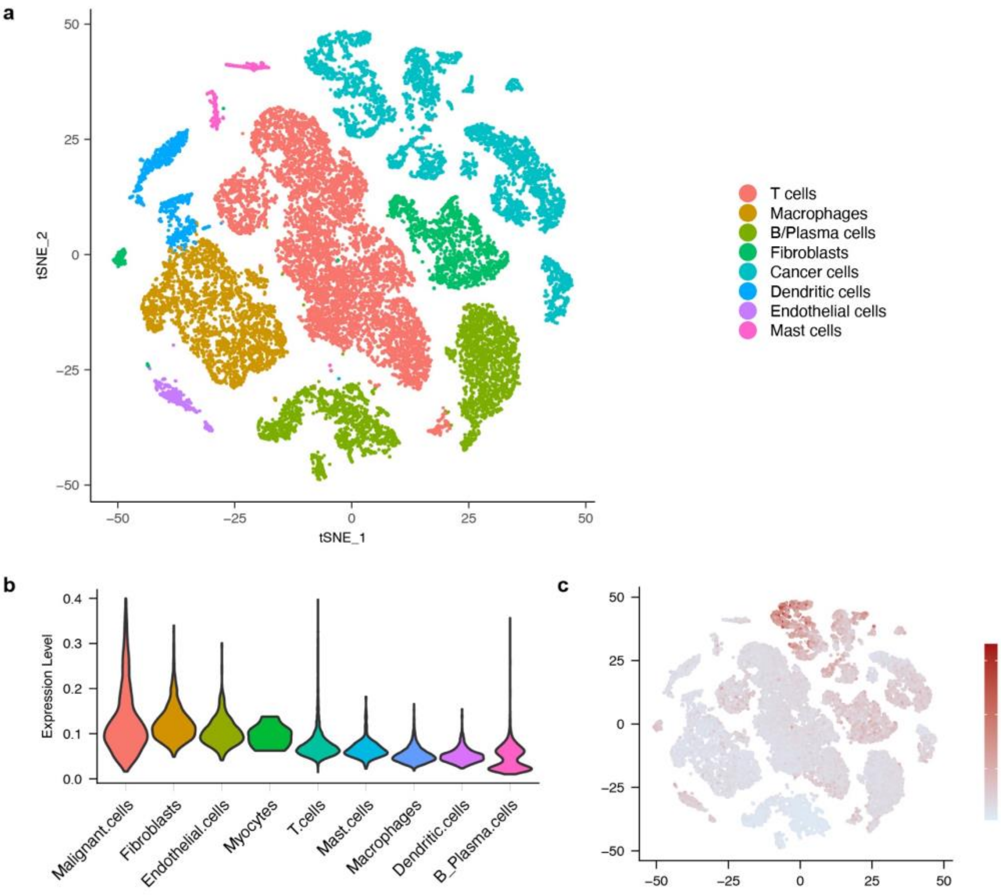

Supplementary figure 5. The single-cell expression profile of *TRMT61A* in the TME. **a**, tSNE plot representing 35,396 cells sorted from GSE181919 dataset analyzed by single-cell RNA sequencing (scRNA-seq). **b-c**, Normalized relative expression of *TRMT61A*.

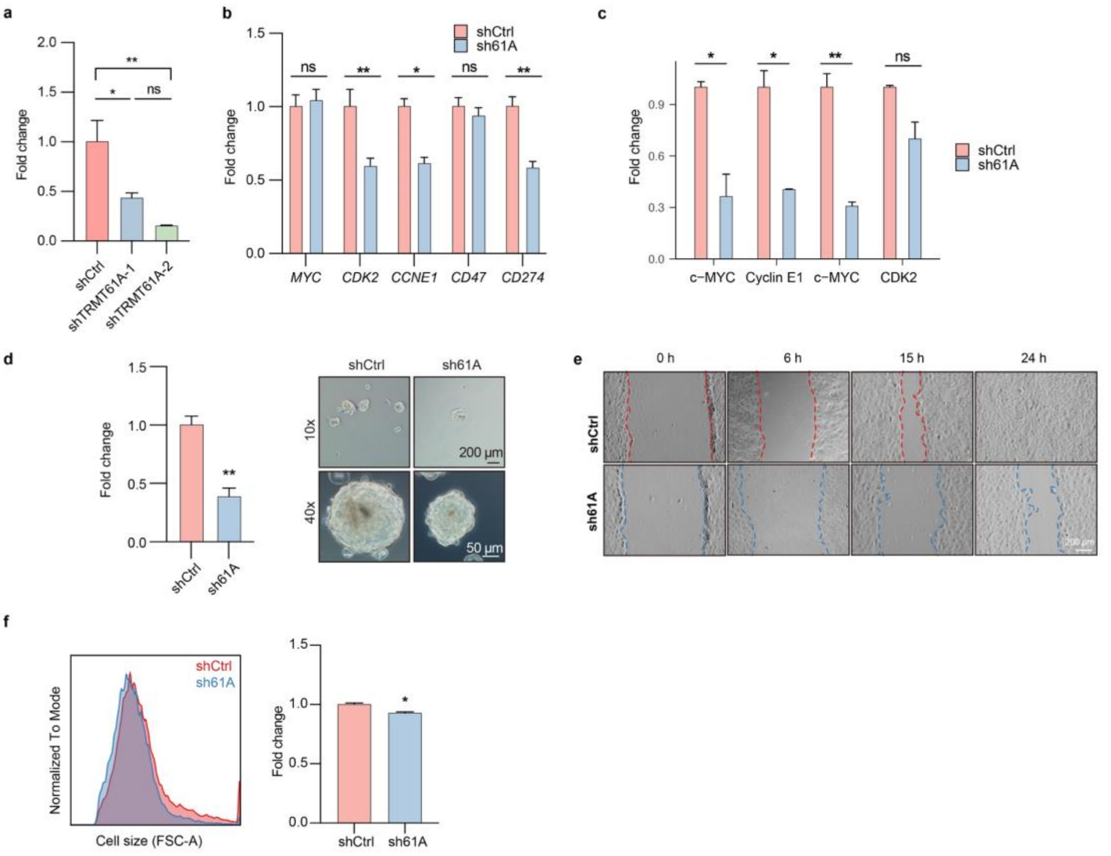

Supplementary figure 6. a-b, mRNA levels in shCtrl/sh61A WSU-HN6 cells were quantified by qPCR (*n* = 3). **c**, Total red-fluorescence integrated density (IntDen) quantified and normalized based on cell numbers for different proteins (*n* = 3). **d**, Sphere formation ability of shCtrl/sh61A WSU-HN6 cells (*n* = 3). Representative pictures are shown to the right. **e**, Representative images from *in vitro* wound healing assays of shCtrl/sh61A WSU-HN6 cells. **f**, Cell sizes of shCtrl/sh61A CAL 27 cells quantified by FSC-A (*n* = 3). Representative flow cytometry histograms are shown to the left. Data are mean with s.e.m. One-way ANOVA followed by Tukey's multiple comparisons tests (**a**). Two-tailed unpaired Student's t-test (**b, d, f**); Two-tailed unpaired Welch's t-test (**c**). *$P < 0.05$; **$P < 0.01$; ns represents no significance.

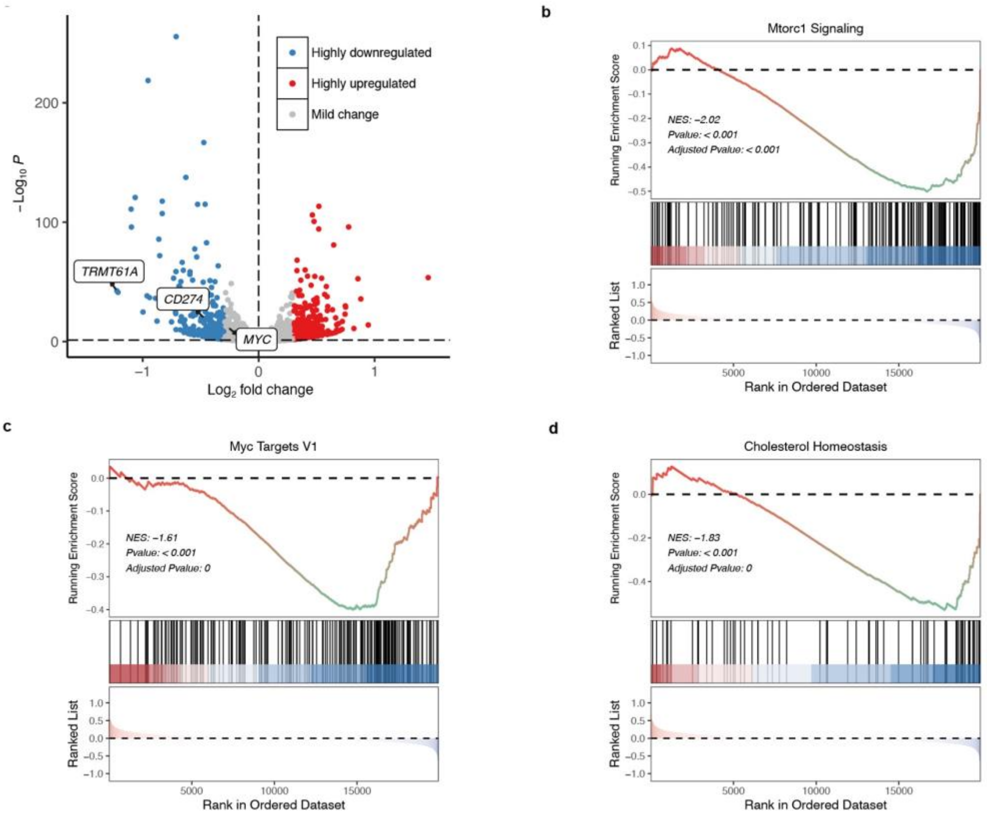

Supplementary figure 7. Bioinformatic analysis of transcriptomic sequencing results of WSU-HN6 cells. **a**, Analysis of high-throughput sequencing results of sh61A WSU-HN6 cells against shCtrl WSU-HN6 cells by a volcano plot (n = 3). **b-d**, Analysis of high-throughput sequencing results of sh61A WSU-HN6 cells against shCtrl WSU-HN6 cells by GSEA (n = 3).

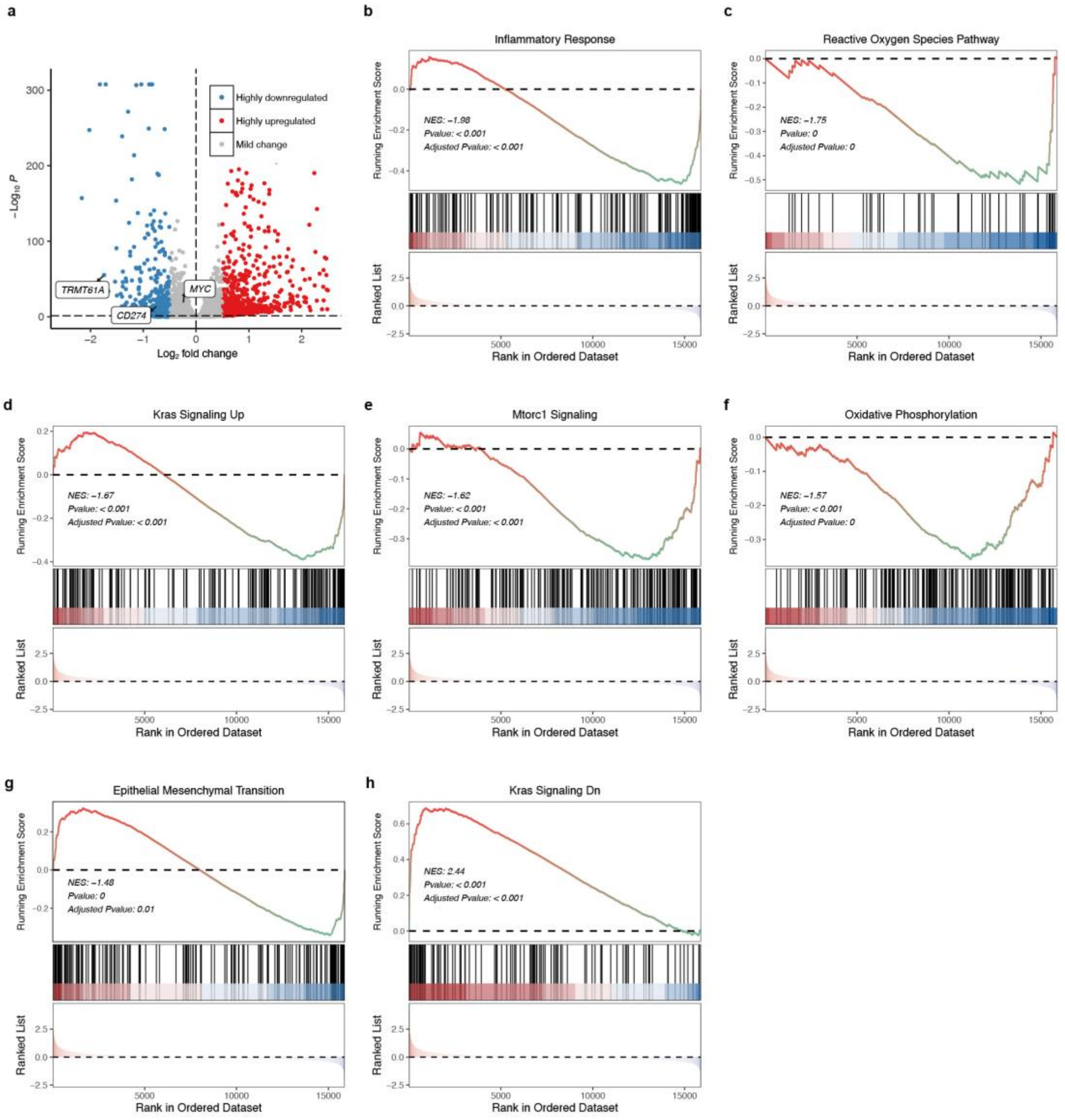

Supplementary figure 8. Bioinformatic analysis of transcriptomic sequencing results of CAL 27 cells. **a**, Analysis of high-throughput sequencing results of sh61A CAL 27 cells against shCtrl CAL 27 cells by a volcano plot (n = 3). **b-h,** Analysis of high-throughput sequencing results of sh61A CAL 27 cells against shCtrl CAL 27 cells by a GSEA (n = 3).

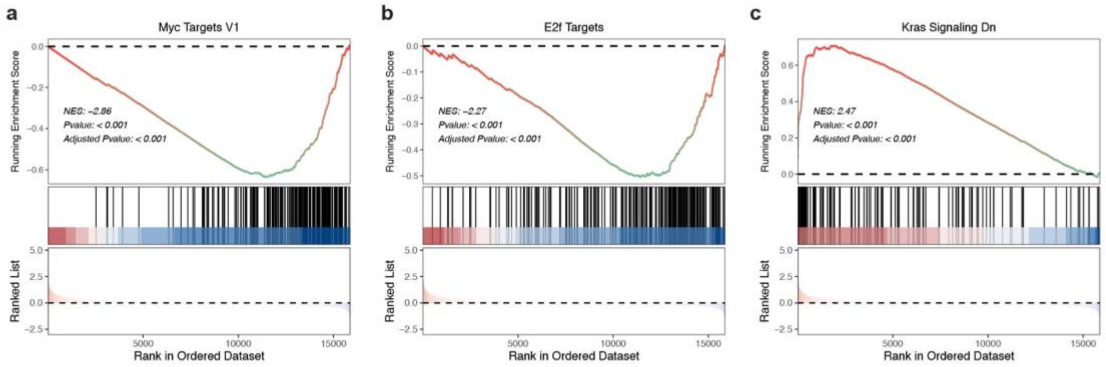

Supplementary figure 9. Bioinformatic analysis of transcriptomic sequencing results of IFNγ-treated CAL 27 cells. **a-c,** Analysis of high-throughput sequencing results of IFNγ-treated sh61A CAL 27 cells against shCtrl CAL 27 cells by GSEA (n = 3).

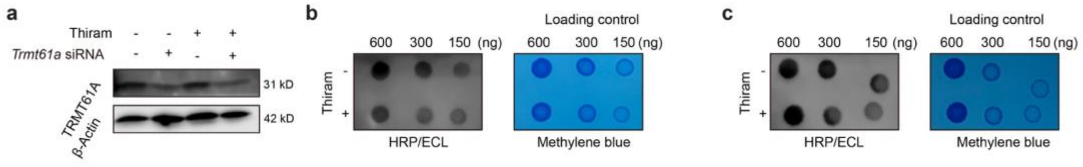

Supplementary figure 10. a, Protein levels of TRMT61A in 4MOSC1 cells treated by thiram and *Trmt61a* siRNA detected by western blotting. **b-c,** Dot blot assay detecting m^1A levels in tumors of 4MOSC1 (**b**) and *Tgfbr1/Pten* 2cKO (**c**) models.

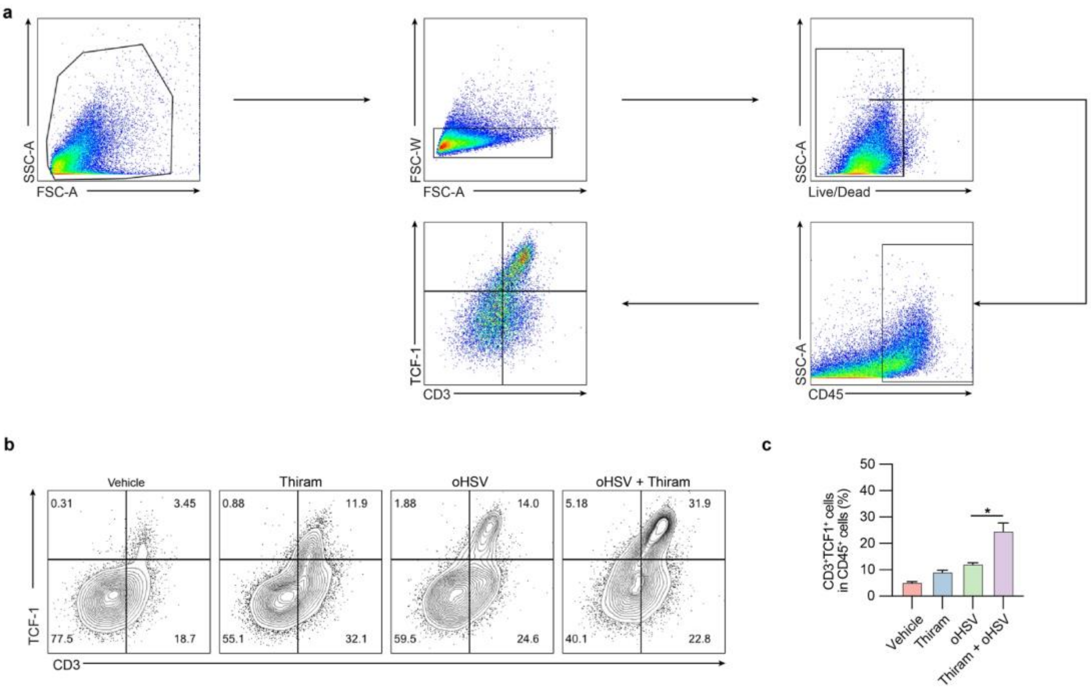

Supplementary figure 11 a, Gating strategy used to identify the $CD3^{+}TCF\text{-}1^{+}$ cells from 4T1 tumors. **b-c**, Representative flow cytometric analysis images (**b**) and quantification (**c**) of CD3Ɛ and TCF-1 expression in the $CD45^{+}$ cells from 4T1 tumors (n = 6 mice per group). Data are mean with s.e.m. *$P < 0.05$ by two-tailed unpaired Welch's t-test.

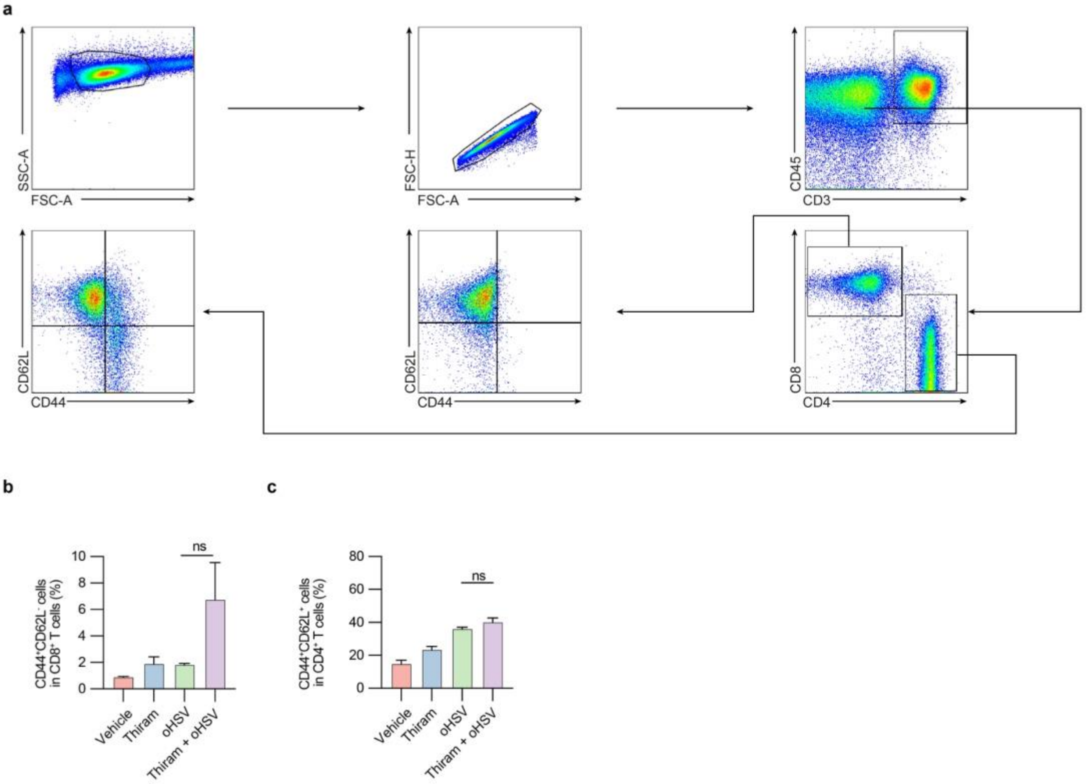

Supplementary figure 12. a, Gating strategy used to identify the CD44$^+$CD62L$^+$ cells from CD4$^+$ or CD8$^+$ T cells from TDLNs of 4MOSC1 tumors. **b-c**, Quantification of CD44 and CD62L expression in the T cells from TDLNs of 4MOSC1 tumors (*n* = 6 mice per group). Data are mean with s.e.m. Two-tailed unpaired Student's t-test (**b-c**). ns represents no significance.

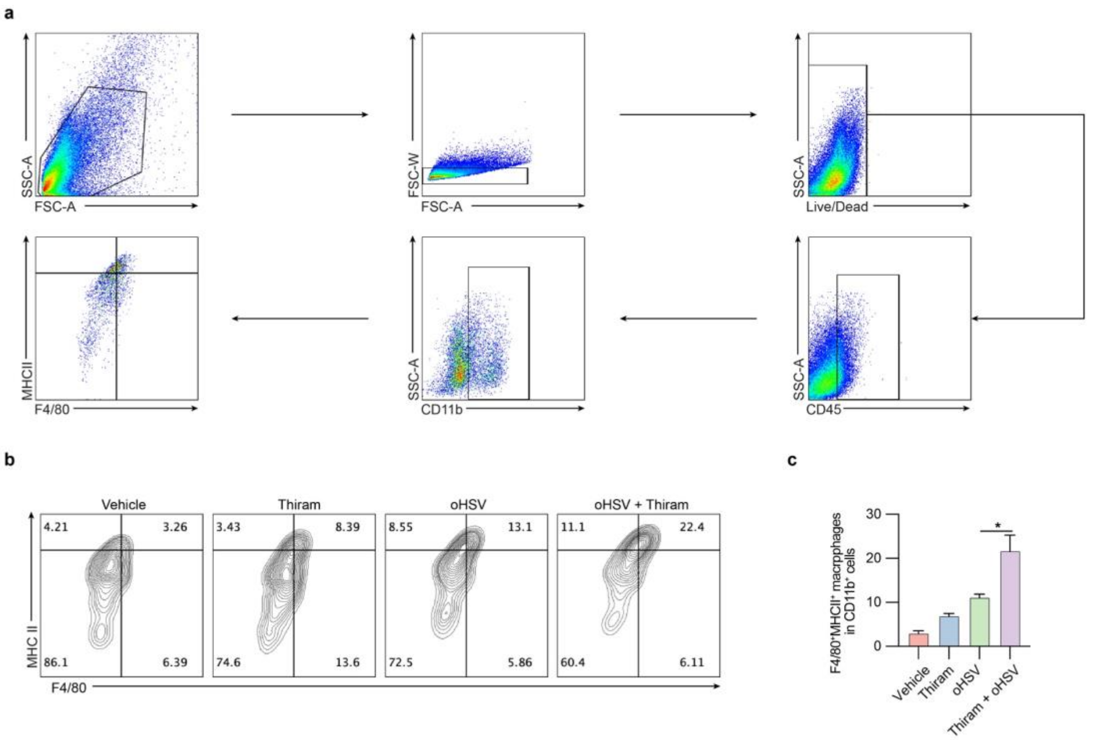

Supplementary figure 13. a, Gating strategy used to identify the F4/80$^+$MHCII$^+$ cells from CD11b$^+$ cells of 4T1 tumors. **b-c**, Representative flow cytometric analysis images (**b**) and quantification (**c**) of F4/80 and MHCII expression in the CD11b$^+$ cells of 4T1 tumors (*n* = 6 mice per group). Data are mean with s.e.m. *$P < 0.05$ by two-tailed unpaired Welch's t-test.

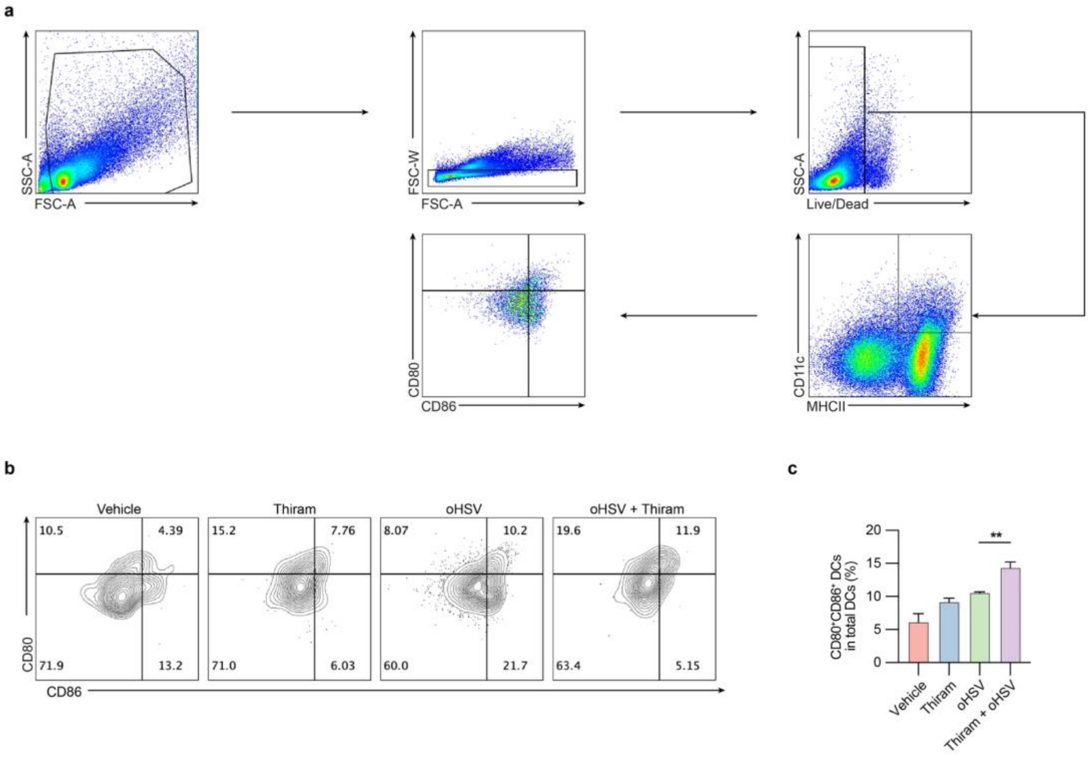

Supplementary figure 14. a, Gating strategy used to identify the CD80^{+}CD86^{+} cells from dendritic cells of 4MOSC1 TDLNs. **b-c**, Representative flow cytometric analysis images (**b**) and quantification (**c**) of F4/80 and MHCII expression in the dendritic cells of 4MOSC1 TDLNs. Data are mean with s.e.m. (n = 6 mice per group). **P < 0.01 by two-tailed unpaired Welch's t-test.

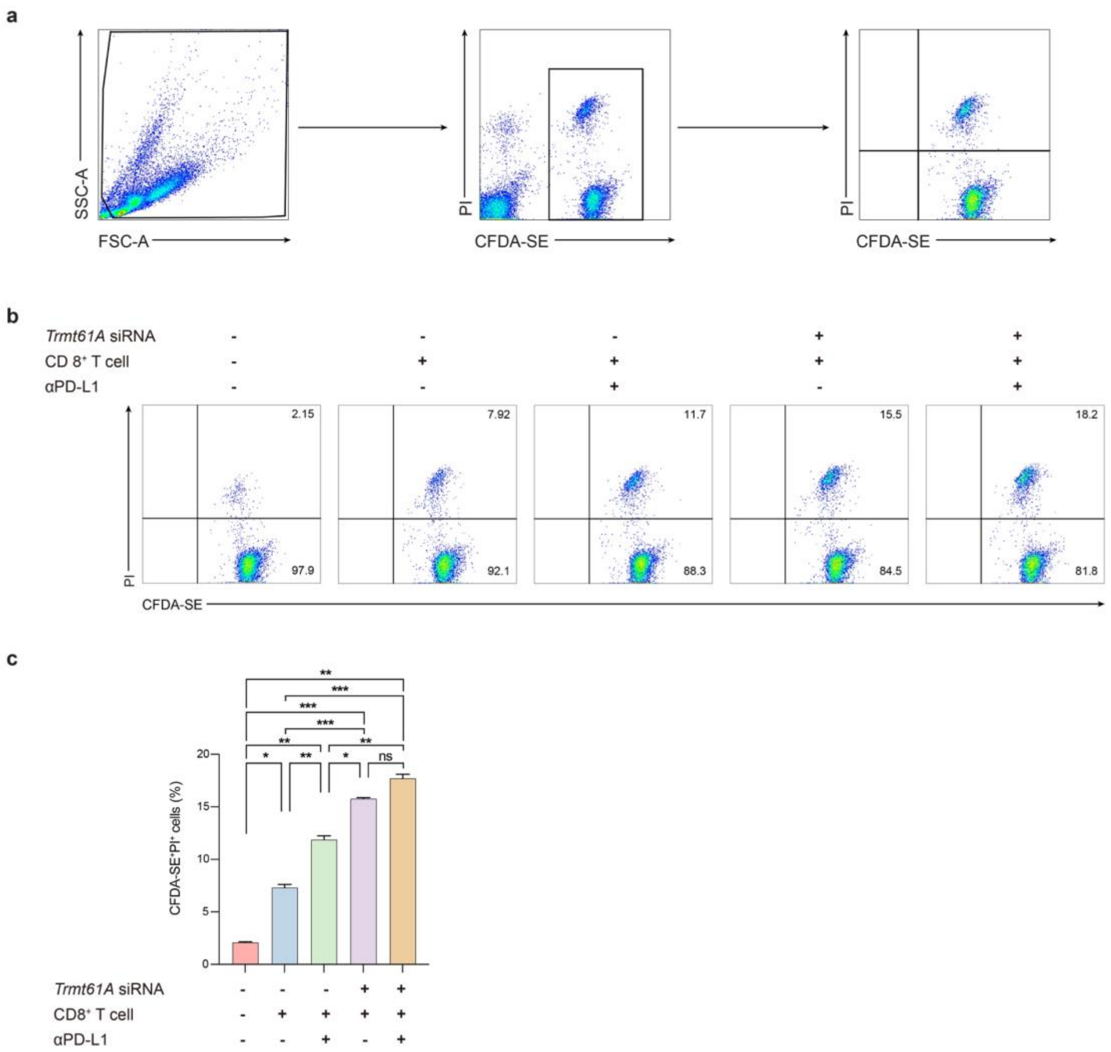

Supplementary figure 15. a, Gating strategy used to identify the CFDA-SE$^+$PI$^+$ 4MOSC1 cells. **b-c**, Representative flow cytometric analysis images (**b**) and quantification (**c**) of CFDA-SE$^+$PI$^+$ 4MOSC1 cells. Data are mean with s.e.m. (n = 3 biological replicates per group). *$P < 0.05$; **$P < 0.01$; ***$P < 0.001$; ns represents no significance by Welch's ANOVA followed by Dunnett's T3 multiple comparisons test.

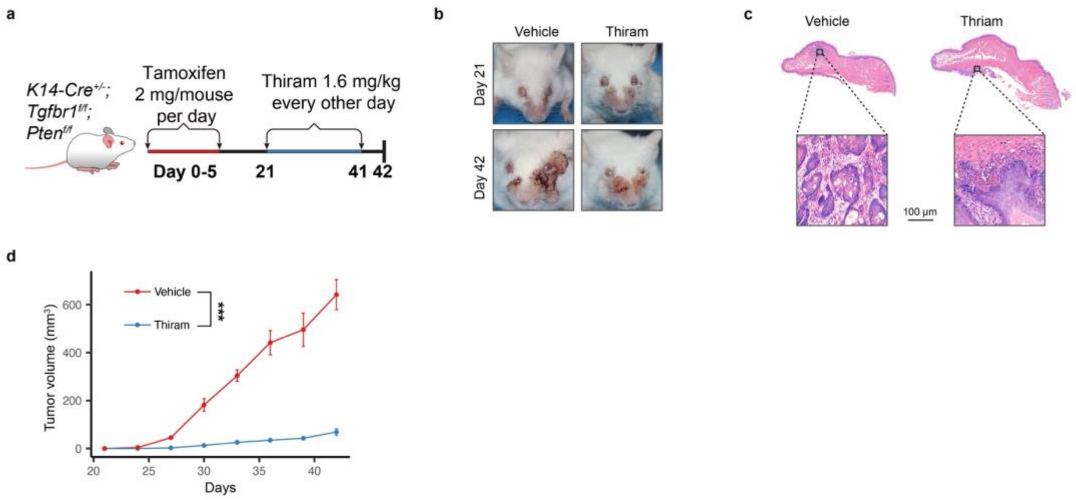

Supplementary figure 16. a-d, Study design (**a**), Representative photograph (**b**), pathological images (**c**), and tumor growth curves (**d**) of HNSCC of the thiram groups and vehicle groups after tamoxifen gavage (n = 6 biological replicates per group). Two-tailed unpaired Student's t-test (**d**). Data are mean with s.e.m. ***$P < 0.001$.

Tissue preparation and flow cytometry. For cultured cells, shCtrl/sh61A cells were trypsinized, washed, and stained with antibodies against PD-L1 (BioLegend, cat. no. 329707), CD47 (BioLegend, cat. no. 323123), CD155 (BioLegend, cat. no. 337617) at 4 °C for 30 min. For immunocompetent allograft mouse models, tumors and tumor-draining lymph nodes were collected, processed into a single-cell suspension using the gentleMACS Dissociator (Miltenyi Biotec), and pressed through a 200-gauge mesh. Antibodies against CD45 (cat. no. 103154), CD11b (cat. no. 101230), F4/80 (cat. no. 123107), MHCII (cat. no. 107608), CD206 (cat. no. 141708), PD-L1 (cat. no. 124315), CD3Ɛ (cat. no. 100306), CD4 (cat. no. 100531), CD8a (cat. no. 100758), TCF-1 (cat. no. 655203), CD44 (cat. no. 103007), CD62L (cat. no. 104412) were purchased from BioLegend. The cells were stained with antibodies (1:200) at 4 °C for 30 min. After surface antibody staining, cells were fixed and permeabilized with a Fixation/Permeabilization Solution Kit (BD Biosciences), followed by staining with antibodies against intracellular molecules. Data were collected with a Beckman CytoFLEX LX flow cytometer (Beckman Coulter, Brea, California, USA) and analyzed in FlowJo (Version 10.0.6, Tree Star).

Real-time PCR. Total RNA was extracted using an RNA purification kit (Axygen, cat. no. AP-MN-MS-RNA). The primer sequences used for real-time PCR are as follows:

TRMT61A: 5'-AGCTTCGTGGCATACGAGG-3', 5'-CCGATAAGGTCAACTGAGTGC-3';
MYC: 5'-GGCTCCTGGCAAAAGGTCA-3', 5'-CTGCGTAGTTGTGCTGATGT-3';
ACTB: 5'-CATGTACGTTGCTATCCAGGC-3', 5'-CTCCTTAATGTCACGCACGAT-3';
CDK2: 5'-CCAGGAGTTACTTCTATGCCTGA-3', 5'-TTCATCCAGGGGAGGTACAAC-3';
CCNE1: 5'-AAGGAGCGGGACACCATGA-3', 5'-ACGGTCACGTTTGCCTTCC-3';
CD47: 5'-AGAAGGTGAAACGATCATCGAGC-3', 5'-CTCATCCATACCACCGGATCT-3';
Cd274: 5'-GCTCCAAAGGACTTGTACGTG-3', 5'-TGATCTGAAGGGCAGCATTTC-3';
Trmt61a: 5'-ATGAGTTTCGTGGCATACGAG-3', 5'-CACGCACTGCTACCATCGAG-3';
Actb: 5'-GGCTGTATTCCCCTCCATCG-3', 5'-CCAGTTGGTAACAATGCCATGT-3'.

Immunohistochemistry and TMA staining. The usage of human samples included in this study was approved by the Institutional Medical Ethics Committee of School and Hospital of Stomatology, Wuhan University (2014LUNSHENZI06, 2016LUNSHENZI62). HNSCC samples were collected from the patients in the Hospital of Stomatology of Wuhan University. The pathological analysis was carried out by two independent pathologists of the Department of Oral Pathology, Wuhan University. All the HNSCC samples were made into human HNSCC

tissue array, including 210 primary HNSCC samples, 69 oral epithelial dysplasia samples, and 42 normal mucosal samples (Outdo Biotech, Shanghai). The custom made human HNSCC tissue microarrays were used in this study as described previously[1].

Paraffin-embedded tumor samples were used for analysis animal tumors. After cutting (Leica RM2235), the slides were baked at 60 ℃ for 20 min for de-paraffinization. Tissues were then treated to unmask antigen. For IHC staining, endogenous peroxidases were inactivated by 3% H_2O_2 at room temperature for 15 min. Then tissues were treated with 5% BSA and 5% goat serum for 1 h to block non-specific signals. Tissues were then stained with primary antibodies against PD-L1 (Abcam, cat. no. ab213524) or CD3ε (Cell signaling technology, cat. no. 99940) according to the manufacturer's instructions. After washing with PBS, tissues were stained with secondary antibodies.

Immunofluorescence staining. Immunofluorescence staining was conducted on shCtrl/sh61A cells. Specifically, the cells were exposed to primary antibodies that targeted distinct markers: c-MYC (Epitomics, cat. no. pro_36033), Cyclin E1 (Abcam, cat. no. ab33911), and CDK2 (Abcam, cat. no. ab32147). Following this, fluorescence-conjugated secondary antibodies (ZSGB-BIO, cat. no. ZF-0516) were employed to facilitate imaging. The resulting fluorescence signals were observed through the use of a confocal microscope (Eclipse Ti, Nikon). For quantitative analysis, the overall integrated density (IntDen) of red fluorescence, calculated using ImageJ software version 1.53k for Mac OS (LOCI, University of Wisconsin), was normalized relative to the cell count.

Bioinformatic analysis of public data. RNA sequencing data of the TCGA-HNSC cohort were download and processed via "TCGAbiolinks" R package. Hallmark gene sets were downloaded from www.gsea-msigdb.org and gene set enrichment analysis (GSEA) analysis were carried out with "clusterProfiler" R package. The HNSC bulk-seq dataset was downloaded from the Gene Expression Omnibus (Series GSE41613)[2]. Single-cell sequencing data were downloaded from the Gene Expression Omnibus (Series GSE181919) and processed with "Seurat" 4.3.0, and cell types are annotated according to the publishers[3].

Western Blot Analysis. Antibodies against TRMT61A (Thermo Fisher Scientific, cat. no. PA5-76553), c-MYC (Epitomics, cat. no. pro_36033), Cyclin E1 (Abcam, cat. no. ab33911), PD-L1 (Abcam, cat. no. ab213524), CDK2 (Abcam, cat. no. ab32147), N-Cadherin (Cell Signaling Technology, cat. no. 13116), E-Cadherin (Cell Signaling

Technology, cat. no. 3195), α-Smooth Muscle Actin (Cell Signaling Technology, cat. no. 19245), Vinculin (Abmart, cat. no. T40106), β-catenin (Abmart, cat. no. M24002), and beta Actin (Abcam, cat. no. ab8226) were diluted in blocking buffer (Beyotime, cat. no. P0252) at concentrations of 1:1000. After electrophoresis, the protein samples were transferred to polyvinylidene difluoride (PVDF) membranes and then incubated at room temperature in 5% skimmed milk for 1 h and overnight in primary antibody solution at 4 ℃. The membranes were then incubated with secondary antibody for 1 h, and an ECL kit (BioSharp, cat. no. BL523A) was used to detect proteins. An Odyssey system (LI-COR Biosciences) was used to visualize protein bands. For protein quantification, Image Studio analysis software (LI-COR Biosciences) was employed.

Dot blot. RNA extraction was carried out using an RNA purification kit (Axygen, cat. no. AP-MN-MS-RNA), then the purified RNA was quantified and diluted in 10 mM Tris-EDTA buffer. The denaturation of equivalent amounts of RNA took place at 65 °C for 5 minutes, after which it was loaded onto a positively charged nylon membrane (Beyotime, cat. no. FFN10). Subsequently, the membrane was subject to UV-crosslinking twice at 2400 J/cm^2, followed by probing with anti-m^{1}A antibody (Abcam, cat. no. ab208196), staining with HRP-conjugated secondary antibody, and ECL detection.

Colony formation assay. In order to measure clonogenicity, cells were seeded at a density of 1000 cells/well in six-well plates and left for one week for colonies to form. Colonies are then fixed, stained with crystal violet to make them visible, and counted.

Codon-switch assay. The coding DNA sequences of *MYC-WT* and *MYC-Mut* were constructed into a pcDNA3.1(+) plasmid. Transfection was performed using Lipofectamine™ 3000 (Invitrogen, USA). Cells were collected for western blot analysis 48 h after transfection.

Sphere formation assay. HNSCC single cells were resuspended in sphere formation medium (DMEM-F12 + 1% B27 supplement + 20 ng/ml bFGF + 20 ng/ml EGF + 1% N2 supplement) and inoculated in ultralow attachment 6-well plates (Corning, cat. no. 3471) at a concentration of 5000 cells/well. Four days later, round cell clusters >100 μm were counted as spheres.

Generation of oncolytic HSV-1 D34.5/D47-IpaH9.8. ICP34.5- and ICP47-deficient HSV-1 (HSV-1 Δ34.5/Δ47; referred to as oHSV in the study) was generated by removing the coding sequences of ICP34.5 and ICP47 in a bacterial artificial chromosome (BAC) using a two-step Red-mediated recombination method. A detailed

description of the procedure can be found in our previous study[4].

Chemicals and reagents. Four canonical nucleoside standards (rA, rU, rG and rC), 11 modified nucleoside standards and one isotopic nucleoside standard (rC-$^{13}C_5$) were purchased from various commercial resources. The CAS numbers, molecular formulas, and molecular weights of these nucleoside standards can be found in Table S1.

Calf intestinal alkaline phosphatase (CIAP) and S1 nuclease were purchased from Takara Biotechnology (Dalian, China). Venom phosphodiesterase I was purchased from Sigma-Aldrich (Beijing, China). Analytical grade ethanol and formic acid (FA) were purchased from Sinopharm Chemical Reagent Co., Ltd. (Shanghai, China). HPLC grade trichloromethane was purchased from Yonghua Chemical Co., Ltd. (Jiangsu, China). LC-MS grade methanol was purchased from FTSCI (Wuhan, China).

Enzymatic digestion of RNA. A 30-μL mixture containing ~3 μg of small RNA, 0.125 U of phosphodiesterase I, 90 U of S1 nuclease, 7.5 U of CIAP and 2 μL of reaction buffer was incubated at 37 °C for 6 h. Then 270 μL of H_2O and 300 μL of chloroform were added followed by vortexing for 3 min and centrifugation at 12000 rpm for 5 min. The digested samples were extracted with chloroform three times. The aqueous layer was lyophilized to dryness and dissolved in water and then subjected to LC-ESI-MS/MS analysis.

LC-MS/MS analysis. Analysis of nucleosides was performed on a Shimadzu 8045 mass spectrometer (Kyoto, Japan) coupled with a Shimadzu LC-30AD UPLC system (Tokyo, Japan) according to previous studies.[5,6] The analysis was conducted under positive ion mode and multiple reaction monitoring (MRM) was employed for the detection. The chromatographic separation of nucleosides was performed on a Shimadzu Shim-pack GIST C18 column (100 mm × 2.1 mm i.d., 2.0 μm) at 40 °C. Solvent A of 0.05% FA/H2O and solvent B of MeOH were used for the chromatographic separation with a flow rate of 0.2 mL min^{-1}. The gradient for the separation was 0−2 min, 5% B; 2−10 min, 5% - 80% B; 10−13 min, 80% B, 13−20 min, 80%−5% B. The MRM parameters of these nucleosides were optimized and the optimized MS parameters are listed in Table S2.

The calibration curves were built to quantitatively measure the nucleosides. Different amounts of nucleoside standards mixed with a given amount of isotopic internal standard (rC-$^{13}C_5$) were utilized to construct the calibration curves by plotting the peak area ratios (nucleosides/rC-$^{13}C_5$) versus the amounts of nucleosides. The amounts of modified nucleosides were calculated and normalized to their corresponding normal nucleosides to

obtain the molar ratio.

Co-Culture Study. The $CD8^+$ T cells are isolated from the spleens of the mice cured by the thiram and oHSV combination therapy in the 4MOSC1 model. A mouse $CD8^+$ cell separation kit (RWD Life Science, USA) was used, and $CD8^+$ T cells were adjusted to 5×10^5 cells/mL in RPMI-1640 medium. Three hours prior to co-culture, $CD8^+$ T cells and the siRNA-transfected 4MOSC1 cells and were stimulated by oHSV at an MOI of 0.1. Then CFDA-SE-labeled 4MOSC1 cells (1×10^5 cells/mL) were plated in 12-well plates at 0.5 mL per well, followed by the addition of 0.5 mL $CD8^+$ T cells (5×10^5 cells/mL) to the 12-well plate with gentle agitation. PD-L1 antibody (10 μg/ml；Bio X Cell, cat. no. BE0101) and thiram (10 μM; Aladdin, cat. no. T111114) are added as indicated. Simultaneously, the target cells were incubated alone to ascertain spontaneous cell death. After a 24-hour incubation, both $CD8^+$ T cells and 4MOSC1 cells were collected and stained with Propidium iodide (PI). The flow cytometry assessment of the death of 4MOSC1 cells (the ratio of $CFDA\text{-}SE^+/PI^+$) was conducted following gating on the CFDA-SE labeled 4MOSC1 cells.

Cell invasion assay. In the invasion assay, transwell chambers (Costar, cat. no. 3422) were utilized with polycarbonate filters coated with 60 µl of basement membrane matrix (1:9, MCE, cat. no. HY-K6002). Subsequently, shCtrl/sh61A WSU-HN6 cells were seeded at a concentration of $2*10^4$/well onto the synthetic basement membrane. Fetal bovine serum was introduced to the lower chamber to act as a chemoattractant. The cells were then allowed to invade through the polycarbonate filters over a 24-hour incubation period at 37˚C. Following incubation, the filters were fixed and stained using a 0.1% crystal violet solution. Non-invading cells were eliminated using a cotton swab, and the invading cells located on the underside of the filter were enumerated using an inverted microscope.

Sulforhodamine B assay. The sulforhodamine B (SRB) assay was carried out as previously reported[7,8]. Briefly, cell monolayers are fixed using 10% (wt/vol) trichloroacetic acid (TCA) and then stained with the SRB dye for 30 minutes. The excess dye is subsequently removed by washing repeatedly with 1% (vol/vol) acetic acid. The protein-bound dye is then dissolved in a 10 mM Tris base solution, and the optical density (OD) is determined at 545 nm using a microplate reader.

1 Liu, Y. *et al.* LIMP-2 enhances cancer stem-like cell properties by promoting autophagy-induced GSK3β degradation in head and neck squamous cell carcinoma. *Int J Oral Sci* **15**, 24, doi:10.1038/s41368-023-00229-0 (2023).

2 Cillo, A. R. *et al.* Immune Landscape of Viral- and Carcinogen-Driven Head and Neck Cancer. *Immunity* **52**, 183-199.e189, doi:10.1016/j.immuni.2019.11.014 (2020).
3 Choi, J. H. *et al.* Single-cell transcriptome profiling of the stepwise progression of head and neck cancer. *Nat Commun* **14**, 1055, doi:10.1038/s41467-023-36691-x (2023).
4 Xie, J. *et al.* Oncolytic herpes simplex virus armed with a bacterial GBP1 degrader improves antitumor activity. *Mol Ther Oncolytics* **29**, 61-76, doi:10.1016/j.omto.2023.04.006 (2023).
5 Chen, M. Y. *et al.* Adolescent alcohol exposure alters DNA and RNA modifications in peripheral blood by liquid chromatography-tandem mass spectrometry analysis. *Chin Chem Lett* **33**, 2086-2090, doi:10.1016/j.cclet.2021.08.094 (2022).
6 Chen, M. Y. *et al.* Comprehensive profiling and evaluation of the alteration of RNA modifications in thyroid carcinoma by liquid chromatography-tandem mass spectrometry. *Chin Chem Lett* **33**, 3772-3776 (2022).
7 Vichai, V. & Kirtikara, K. Sulforhodamine B colorimetric assay for cytotoxicity screening. *Nat Protoc* **1**, 1112-1116, doi:10.1038/nprot.2006.179 (2006).
8 Skehan, P. *et al.* New colorimetric cytotoxicity assay for anticancer-drug screening. *J Natl Cancer Inst* **82**, 1107-1112, doi:10.1093/jnci/82.13.1107 (1990).

Table S1

Information of the 16 nucleoside standards.

No	Nucleosides	Abbreviation	CAS number	Molecular formula	Molecular weight	Company
**1**	adenosine	rA	58-61-7	$C_{10}H_{13}N_5O_4$	267.24	Sigma-Aldrich
**2**	guanosine	rG	118-00-3	$C_{10}H_{13}N_5O_5$	283.24	Sigma-Aldrich
**3**	cytidine	rC	65-46-3	$C_9H_{13}N_3O_5$	243.22	Sigma-Aldrich
**4**	uridine	rU	58-96-8	$C_9H_{12}N_2O_6$	244.2	Sigma-Aldrich
**5**	cytidine-1',2',3',4',5'-$^{13}C_5$	rC-$^{13}C_5$	-	$C_4{}^{13}C_5H_{13}N_3O_5$	248.18	CATO
**6**	N^1-methyladenosine	m^1A	15763-06-1	$C_{11}H_{15}N_5O_4$	281.27	J&K Scientific Ltd.
**7**	N^6-methyladenosine	m^6A	1867-73-8	$C_{11}H_{15}N_5O_4$	281.27	Hanhong Chemical
**8**	N^6,2'-*O*-dimethyladenosine	m^6Am	57817-83-1	$C_{12}H_{17}N_5O_4$	295.29	Granlen
**9**	N^6,N^6-dimethyladenosine	$m^{6,6}A$	2620-62-4	$C_{12}H_{17}N_5O_4$	295.29	J&K Scientific Ltd.
**10**	2'-*O*-methyladenosine	Am	2140-79-6	$C_{11}H_{15}N_5O_4$	281.27	J&K Scientific Ltd.
**11**	N^2,N^2,7-trimethylguanosine	$m^{2,2,7}G$	40027-70-1	$C_{13}H_{19}N_5O_5$	325.32	Carbosynth
**12**	2'-*O*-methylguanosine	Gm	2140-71-8	$C_{11}H_{15}N_5O_5$	297.27	Carbosynth
**13**	N^4-acetylcytidine	ac^4C	3768-18-1	$C_{11}H_{15}N_3O_6$	285.25	Carbosynth
**14**	2'-*O*-methyluridine	Um	2140-76-3	$C_{10}H_{14}N_2O_6$	258.23	Carbosynth
**15**	3-methyluridine	m^3U	2140-69-4	$C_{10}H_{14}N_2O_6$	258.23	Carbosynth
**16**	pseudouridine	Y	1445-07-4	$C_9H_{12}N_2O_6$	244.2	Carbosynth

Table S2

The optimized MRM parameters for analysis of nucleosides by LC-MS/MS.

**Nucleosides**	**Precursor ion (*m/z*)**	**Product ion (*m/z*)**	**Q1 (V)**	**CE (V)**	**Q3(V)**	**RT (min)**
rA	268.1	136.1	-	-65	-	4.259
rG	284.1	152	-	-50	-	4.34
rC	244.1	112.1	-	-53	-	1.745
rU	245.1	113	-	-35	-	2.76
rC-$^{13}C_5$	249.2	112.1	-	-53	-	1.759
m^1A	282.1	150.1	-11	-18	-29	1.987
m^6A	282.1	150.1	-11	-19	-26	6.176
m^6Am	296.1	150.1	-11	-18	-24	7.055
$m^{6,6}A$	296.1	164.1	-24	-20	-16	2.78
Am	282.1	136.1	-12	-18	-24	5.738
$m^{2,2,7}G$	326.1	194.1	-26	-15	-13	5.557
Gm	298.1	152.1	-23	-23	-26	5.77
ac^4C	286.1	154.1	-12	-10	-16	5.986
Um	259.1	113.1	-21	-10	-20	5.359
m^3U	259.1	127.1	-10	-10	-12	5.548
Y	245.2	125.2	-13	-19	-25	1.937

Table S3

Analytes	Linear range	Calibration curve data			LOD	LOQ
		**Slope**	**Intercept**	R^2 **value**	**(fmol)**	**(fmol)**
rA	2-5000 (pmol)	3.733×10^{-2}	-45.9	0.992	-	-
rG	5-5000 (pmol)	3.725×10^{-2}	-36.43	0.9933	-	-
rC	2-5000 (pmol)	2.542×10^{-2}	-27.27	0.9999	-	-
rU	5-1000 (pmol)	1.139×10^{-2}	-32.87	0.9934	-	-
m^{1}A	20-2000 (fmol)	1.656×10^{-2}	1.405×10^{-2}	0.995	0.38	1.25
m^{6}A	2-2000 (fmol)	1.249×10^{-2}	-23.7	0.9919	0.25	0.85
m^{6}Am	2-2000 (fmol)	1.008×10^{-2}	-22.8	0.999	0.33	1
m^{6,6}A	1-500 (fmol)	1.732×10^{-2}	-86.14	0.9971	0.1	0.33
Am	2-5000 (fmol)	7.360×10^{-3}	8.420×10^{-3}	0.9954	0.33	1
m^{2,2,7}G	2-2000 (fmol)	2.237×10^{-2}	-34.6	0.9965	0.05	0.17
Gm	20-20000 (fmol)	2.140×10^{-3}	-17.06	0.9998	1	3.33
ac^{4}C	10-5000 (fmol)	1.460×10^{-3}	-55.4	0.9993	0.33	1
Um	00-500000 (fmo	1.187×10^{-4}	-51.21	0.9984	21.82	72.73
m^{3}U	20-5000 (fmol)	8.202×10^{-4}	-65.8	0.9951	2.5	8.33
Y	000-500000 (fm	9.538×10^{-6}	1.529×10^{-2}	0.9903	200	666

Table S4

Association of TRMT61A expression and clinicopathologic parameters in HNSCC

**Parameters**	**Medians/Mean rank, *n***	***P* value/Adjusted *P* value**
Tissue		
Mucosa	108.3, *n* = 42	**Mucosa vs. HNSCC < 0.0001*** **
Dysplasia	139.8, *n* = 69	Dysplasia vs. Mocosa = 0.2463
HNSCC	178.5, *n* = 210	**HNSCC vs. Dysplasia = 0.0080** **
Age (years)		
< 55	191.8, *n* = 91	0.1974
≥ 55	208.2, *n* = 119	
Sex		
Male	198.3, *n* = 164	0.1033
Female	216.9, *n* = 46	
Tumor Size		
T1 + T2	199.5, *n* = 145	0.3540
T3 + T4	217.8, *n* = 65	
Tumor Grade		
I	90.72, *n* = 53	I vs. II = 0.0675
II	113.6, *n* = 121	I vs. III > 0.9999
III	100.2, *n* = 36	II vs. III = 0.7406
Lymph node status		
N0	188.5, *n* = 138	**0.0016** **
N1 + N2	223.6, *n* = 72	
Smoking		
Yes	208.4, *n* = 123	0.1924
No	197.3, *n* = 87	
Drinking		
Yes	205.0, *n* = 102	0.602
No	200.4, *n* = 108	

Comparisons between groups were performed using the Mann-Whitney U test (2 groups) or Kruskal-Wallis test followed by Dunn's test (> 2 groups).
